# Contrasting responses of above- and belowground diversity to multiple components of land-use intensity

**DOI:** 10.1038/s41467-021-23931-1

**Published:** 2021-06-24

**Authors:** Gaëtane Le Provost, Jan Thiele, Catrin Westphal, Caterina Penone, Eric Allan, Margot Neyret, Fons van der Plas, Manfred Ayasse, Richard D. Bardgett, Klaus Birkhofer, Steffen Boch, Michael Bonkowski, Francois Buscot, Heike Feldhaar, Rachel Gaulton, Kezia Goldmann, Martin M. Gossner, Valentin H. Klaus, Till Kleinebecker, Jochen Krauss, Swen Renner, Pascal Scherreiks, Johannes Sikorski, Dennis Baulechner, Nico Blüthgen, Ralph Bolliger, Carmen Börschig, Verena Busch, Melanie Chisté, Anna Maria Fiore-Donno, Markus Fischer, Hartmut Arndt, Norbert Hoelzel, Katharina John, Kirsten Jung, Markus Lange, Carlo Marzini, Jörg Overmann, Esther Paŝalić, David J. Perović, Daniel Prati, Deborah Schäfer, Ingo Schöning, Marion Schrumpf, Ilja Sonnemann, Ingolf Steffan-Dewenter, Marco Tschapka, Manfred Türke, Juliane Vogt, Katja Wehner, Christiane Weiner, Wolfgang Weisser, Konstans Wells, Michael Werner, Volkmar Wolters, Tesfaye Wubet, Susanne Wurst, Andrey S. Zaitsev, Peter Manning

**Affiliations:** 1 Senckenberg Biodiversity and Climate Research Centre (SBIK-F), Senckenberg Gesellschaft für Naturforschung, Frankfurt, Germany; 2 Thünen Institute of Biodiversity, Braunschweig, Germany; 3 Functional Agrobiodiversity, Department of Crop Sciences, University of Göttingen, Göttingen, Germany; 4 Institute of Plant Sciences, University of Bern, Bern, Switzerland; 5 Systematic Botany and Functional Biodiversity, Institute of Biology, Leipzig University, Leipzig, Germany; 6 Plant Ecology and Nature Conservation Group, Wageningen University & Research, Wageningen, The Netherlands; 7 Institute of Evolutionary Ecology and Conservations Genomics, University of Ulm, Ulm, Germany; 8 Department of Earth and Environmental Sciences, The University of Manchester, Manchester, UK; 9 Department of Ecology, Brandenburg University of Technology, Cottbus, Germany; 10 Biodiversity and Conservation Biology, WSL Swiss Federal Research Institute, Birmensdorf, Switzerland; 11 Institute of Zoology, Terrestrial Ecology, University of Cologne, Köln, Germany; 12 UFZ-Helmholtz Centre for Environmental Research, Department of Soil Ecology, Halle (Saale), Germany; 13 German Centre for Integrative Biodiversity Research (iDiv) Halle-Jena-Leipzig, Leipzig, Germany; 14 Animal Ecology I, University of Bayreuth, Bayreuth, Germany; 15 Bayreuth Center for Ecology and Environmental Research (BayCEER), University of Bayreuth, Bayreuth, Germany; 16 School of Natural and Environmental Sciences, Newcastle University, Newcastle Upon Tyne, UK; 17 Forest Entomology, WSL Swiss Federal Research Institute, Birmensdorf, Switzerland; 18 Department of Environmental Systems Science, Institute of Terrestrial Ecosystems, ETH Zürich, Universitätstr. 16, Zürich, Switzerland; 19 Institute of Agricultural Sciences, Department of Environmental Systems Science, ETH Zürich, Universitätstr. 2, Zürich, Switzerland; 20 Department of Landscape Ecology and Resources Management, Justus Liebig University Giessen, Gießen, Germany; 21 Department of Animal Ecology and Tropical Biology, Biocenter, University of Würzburg, Würzburg, Germany; 22 Ornithology, Natural History Museum Vienna, Vienna, Austria; 23 Leibniz Institute DSMZ-German Collection of Microorganisms and Cell Cultures, Braunschweig, Germany; 24 Department of Animal Ecology, Justus Liebig University Giessen, Giessen, Germany; 25 Ecological Networks, Biology, Technische Universität Darmstadt, Darmstadt, Germany; 26 Agroecology, Department of Crop Sciences, Georg-August University of Göttingen, Göttingen, Germany; 27 Institute of Zoology, General Ecology, University of Cologne, Köln (Cologne), Germany; 28 Institute of Landscape Ecology, University of Münster, Münster, Germany; 29 Max Planck Institute for Biogeochemistry, Jena, Germany; 30 Institute of Ecology, Friedrich-Schiller-University Jena, Jena, Germany; 31 DPI Agriculture, NSW Department of Primary Industries, Australian Cotton Research Institute, Narrabri, NSW Australia; 32 Institute of Biology, Functional Biodiversity, Freie Universität Berlin, Berlin, Germany; 33 Terrestrial Ecology Research Group, Department of Ecology and Ecosystem Management, Technical University of Munich, Freising, Germany; 34 Department of Biosciences, Swansea University, Swansea, UK; 35 Department of Community Ecology, UFZ-Helmholtz Centre for Environmental Research, Halle (Saale), Germany; 36 Institute of Ecology and Evolution, Russian Academy of Sciences, Moscow, Russia

**Keywords:** Biodiversity, Community ecology, Grassland ecology

## Abstract

Land-use intensification is a major driver of biodiversity loss. However, understanding how different components of land use drive biodiversity loss requires the investigation of multiple trophic levels across spatial scales. Using data from 150 agricultural grasslands in central Europe, we assess the influence of multiple components of local- and landscape-level land use on more than 4,000 above- and belowground taxa, spanning 20 trophic groups. Plot-level land-use intensity is strongly and negatively associated with aboveground trophic groups, but positively or not associated with belowground trophic groups. Meanwhile, both above- and belowground trophic groups respond to landscape-level land use, but to different drivers: aboveground diversity of grasslands is promoted by diverse surrounding land-cover, while belowground diversity is positively related to a high permanent forest cover in the surrounding landscape. These results highlight a role of landscape-level land use in shaping belowground communities, and suggest that revised agroecosystem management strategies are needed to conserve whole-ecosystem biodiversity.

## Abstract (web summary)

Land use intensification is a major driver of biodiversity change. Here the authors measure diversity across multiple trophic levels in agricultural grassland landscapes of varying management, finding decoupled responses of above- and belowground taxa to local factors and a strong impact of landscape-level land use.

## Introduction

Agricultural landscapes are undergoing dramatic declines in their biodiversity worldwide^1–4^. While the main cause of these declines is broadly attributed to land-use change and land-use intensification^5–7^, previous studies have mostly focused on the response of aboveground biodiversity to a few components of land use, such as the use of pesticides^8^, fertilization intensity^9–11^ or landscape simplification^12–14^. This focus on particular taxa and components of land use fails to capture the complexity of biodiversity responses to land-use intensification, as a wide range of land-use components act simultaneously and at different temporal and spatial scales^15,16^. While components of local-scale land-use intensity, such as fertilization and grazing intensity, may act as filters on local diversity, larger-scale processes may also shape biodiversity via metacommunity processes linked to species dispersal capacities^17–19^. For example, the spill-over of dispersing organisms from nearby or connected high-quality habitats can maintain species populations at sites that would otherwise not support them^17,19–21^. In the context of agricultural landscapes, negative impacts of local land-use intensification might therefore be buffered by the presence of permanent habitats in the surrounding landscape, which act as sources of recolonization^7,22,23^.

Different taxonomic and functional groups are also likely to have contrasting responses to different components of land-use intensification^5,24,25^, but a synthetic view of these differences is lacking. For example, it is clear that above- and belowground diversity respond to distinct drivers at the global scale^26–28^ but the effects of agricultural intensification on belowground biodiversity are relatively poorly understood, and responses may differ to those of aboveground groups, and among different soil organism groups^29–32^. While highly mobile aboveground trophic groups, such as birds and many insects, are widely known to respond to changes in landscape composition and configuration (e.g. the amount of semi-natural habitat in the landscape and its spatial arrangement)^7,16,33,34^, little is known about how alterations to such landscape features affect the diversity of less mobile belowground taxa^35–37^. Soil organisms, especially microbes, were traditionally assumed to be so abundant and generalist that dispersal limitation does not influence their local diversity^38^. Instead, belowground communities are often assumed to be predominantly structured by local soil conditions^31,39,40^ and, in agroecosystems, by local-level land-use intensity, such as fertilization, grazing and tillage regimes^25,41,42^. If belowground communities are shaped by dispersal processes, then the factors driving habitat quality and connectivity may differ from those aboveground. For instance, instead of the linear features, which allow animal movement between habitat patches^43^, it may be the quantity of historically undisturbed soil habitat in the landscape that determines recolonization rates of wind dispersed species^44–47^, as untilled soils foster more abundant and diverse soil communities^31,48^.

**Fig. 1:**
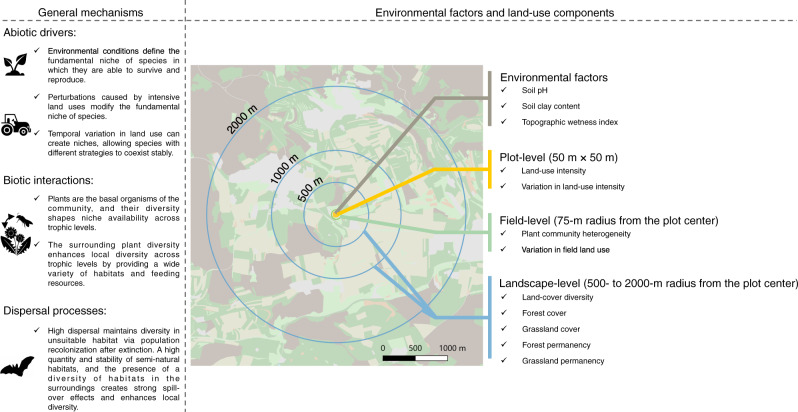
General mechanisms driving the species richness of above- and belowground diversity, and the associated environmental factors and land-use components used in this study. The hypotheses associated with each factor are detailed in the introduction and in the Supplementary Table 1. This figure is not comprehensive but presents a selection of mechanisms, which support the use of the environmental factors and land-use components as predictors. Note also that these expectations are formulated for agroecosystems undergoing anthropogenic disturbances. The categories considered for the general mechanisms were adapted from metacommunity theory^19^. For simplicity, we separate abiotic and biotic drivers, although we acknowledge that abiotic conditions influence species interactions in nature.

The lack of a comprehensive and comparative assessment of how different aspects of local- and landscape-level land use affect above- and belowground trophic groups precludes a holistic understanding of the key drivers of community-level biodiversity loss. Here, we address this gap by using a comprehensive biodiversity and land-use dataset from the German Biodiversity Exploratories project^49^ to compare the influence of multiple land-use components, operating at a range of spatial and temporal scales, on above- and belowground biodiversity in agricultural grasslands. We measure the diversity of ten aboveground trophic groups (primary producers, fungal pathogens, molluscan herbivores, insect herbivores, avian herbivores, insect pollinators, molluscan omnivores, arthropod omnivores, arthropod predators and vertebrate predators), and ten belowground trophic groups (arbuscular mycorrhizal (AM) fungal symbionts, fungal pathogens, fungal decomposers, bacterial decomposers, protistan bacterivores, protistan parasites, protistan omnivores, insect herbivores, arthropod decomposers and arthropod predators). Together, these comprise more than 4000 plant, animal and microbial taxa involved in the delivery of essential agroecosystem services, including nutrient recycling, pollination and biological control^50^. These measures were taken in 150 agricultural grassland fields, that were selected to vary strongly across the full range of local land-use intensity^51^, and which are situated in landscapes of varying complexity^14^ and management history (see Methods). We then test for the associations between the taxonomic diversity of each above- and belowground trophic group and different land-use components. We interpret these associations as evidence of land-use effects and for simplicity we use terms such as ‘effects’ and ‘drivers’ hereafter. While we acknowledge the correlational and static nature of our study, we believe our interpretation is supported by existing knowledge on land-use effects on organisms and the nature of our study design, which minimizes confounding factors (Fig. 1, see also Supplementary Table 1). The land-use components encompass current land-use intensity and land-use history, across three different spatial scales: plot-level factors (i.e. 50 m × 50 m), field-level factors describing the plot surroundings (i.e. 75-m radius from the plot center) and landscape-level factors (i.e. 500–2000-m radii from the plot center) (Fig. 1). In our modelling approach we also account for hypothetically important environmental factors (i.e. soil pH^52–54^, soil clay content^55,56^ and topographic wetness index^57,58^), that are related to potential drivers of niche differentiation and thus species richness (Fig. 1, see also Supplementary Table 1 for specific hypotheses and references).

The variables we relate to trophic group diversities at each of the three scales of organization are selected to represent factors that influence species survival and dispersal. At the plot level, we test for the effect of plot-level land-use intensity, measured as a compound index of grazing, mowing and fertilization intensities^51,59^. These factors, which operate in tandem in agricultural grasslands, alter resource availability and create disturbances, that alter habitat structure, thus altering fundamental co-existence mechanisms such as niche partitioning^29,60^. Local- and field-scale land-use intensity can also vary across time, and this temporal variation in environmental conditions can create niches, allowing species with different strategies to coexist^5^. At the field level, a higher heterogeneity in plant species identities and abundances (i.e. plant community heterogeneity) can maintain local biodiversity by providing a wide variety of habitats and feeding resources, and also buffer local biodiversity loss against environmental changes via small scale dispersal and recolonization^61^. Finally, at larger spatial scales, the conversion of natural or semi-natural habitats, such as forests or grasslands, into agricultural land has been shown to strongly affect local biodiversity and the overall species pool available for recolonization^7^. We therefore consider the effects of the quantity (i.e. forest and grassland cover) and stability (i.e. forest and grassland permanency) of semi-natural habitats, and the presence of a diversity of habitats (i.e. land-cover diversity) in the surrounding landscape, which can significantly affect local biodiversity by creating strong spill-over effects^7,14,22^. There are large differences in resource and habitat requirements, and in movement and dispersal abilities, between the trophic groups considered in this study. Therefore, the impact of each of these components, and the mechanisms through which they operate, is expected to differ in strength and spatial scale between trophic groups (see Supplementary Table 1 for details). Because of this variation, landscape-level land use is characterized for three radii to identify the spatial scale that best explain the species richness of each group (Fig. 1). Three competing models are fitted for each trophic group with the landscape land-use factors calculated either in a 500-m radius, 1000-m radius or 2000-m radius of the grassland plot, and we then select the best model based on the second-order Akaike information criterion (AICc) (Supplementary Table 2). We reveal contrasting responses of above- and belowground biodiversity to land-use intensification, in which local land-use intensity strongly and negatively impacts aboveground trophic groups, but has positive or neutral effects on belowground trophic groups. Our results also highlight a previously unrecognized role of landscape factors in shaping belowground communities, providing strong evidence to support the idea that dispersal processes play a key role in shaping belowground communities. Overall, these findings suggest that a broader view is required in conservation and ecosystem management strategies if we are to preserve both above- and belowground biodiversity, and the vital services they each provide.

## Results and discussion

**Fig. 2:**
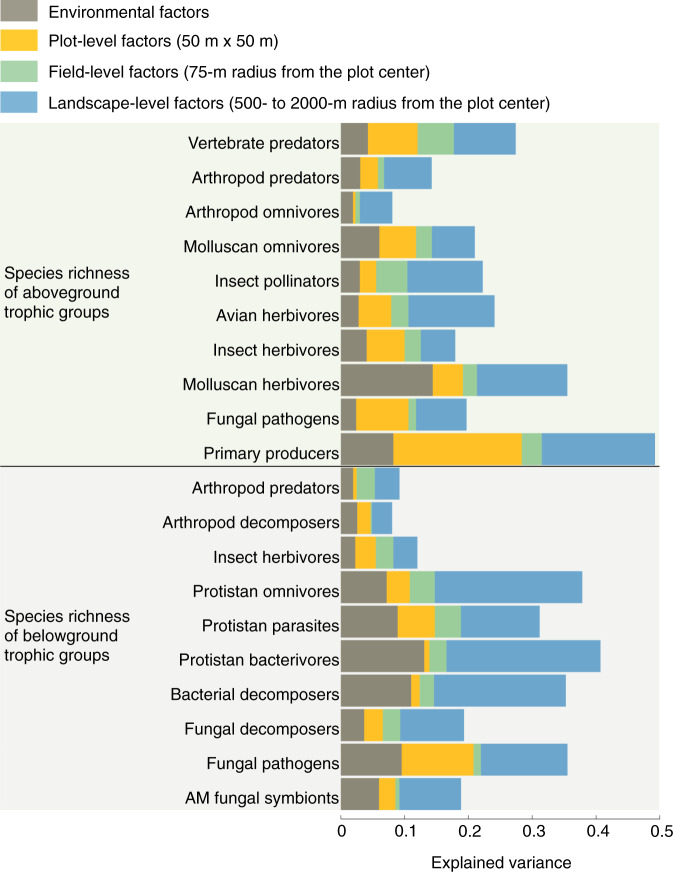
Relative importance of land-use factors in explaining the species richness of multiple above- and belowground trophic groups. Explained variance was calculated for each group of predictors: environmental factors in grey, plot-level (50 m × 50 m) factors in yellow, field level (75-m radius from the plot center) factors in green, and landscape-level (500–2000 m from the plot center) factors in blue. Note that the scale at which landscape land-use factors operate varies among trophic groups (Fig. 4 and Supplementary Table 2). All predictors and response variables were scaled to interpret parameter estimates on a comparable scale.

The species richness of both above- and belowground communities was strongly affected by plot-, field- and landscape-level land use. However, the variance in diversity explained by each scale of land-use component, and their relative importance varied greatly between trophic groups (Fig. 2). In general, explained variance was higher for lower trophic levels (i.e. belowground fungal pathogens, bacterial decomposers, protistan groups, and aboveground primary producers and herbivores) than for higher trophic levels (Fig. 2). Plot-level land-use intensity was an important driver of the species richness of above- and belowground trophic groups, accounting for 24.1% ± 4.0 s.e.m of the explained variance for aboveground trophic groups, and 15.3% ± 3.3 s.e.m of explained variance for belowground trophic groups (Fig. 2). Field-level factors, i.e. plant community heterogeneity and temporal variation in field land use, played a smaller, but significant role, accounting for 11.4% ± 1.9 s.e.m and 11.4% ± 3.0 s.e.m of the variance in aboveground and belowground trophic groups, respectively. Meanwhile, landscape-level land use accounted for the largest proportion of explained variance in both aboveground (43.8% ± 3.6 s.e.m) and belowground trophic groups (47.3% ± 3.3 s.e.m). These results, and those presented below were robust to methodological choices. Sensitivity analyses including the use of raw data instead of region-corrected residuals (Supplementary Figs. 1, 2), the sub-setting of data to exclude plots with overlapping landscape radii (Supplementary Fig. 3) and the use of interaction terms did not affect our overall conclusions (Supplementary Fig. 4).

### Plot-level drivers

**Fig. 3:**
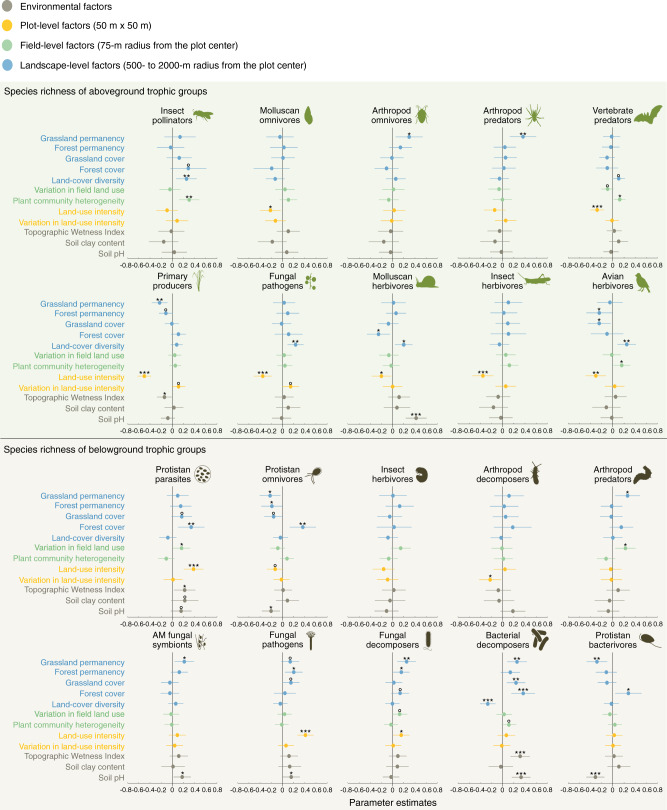
Drivers of the species richness of multiple above- and belowground trophic groups. Data are presented as the parameter estimates (standardized regression coefficients) from linear models and we show the 95% confidence intervals associated with the parameter estimates. Grey points show the parameter estimates of each environmental factor. Yellow points show the parameter estimates of plot-level factors, green points show the parameter estimates of field-level factors; and blue points show the parameter estimates of landscape-level land-use factors. Note that the scale at which landscape land-use factors varies among trophic groups (see Fig. 4 and Supplementary Table 2). All predictors were scaled to interpret parameter estimates on a comparable scale. Plot-level and landscape-level predictors were log-transformed. *P*-values of the best selected models for each model parameter are given as: °*P* < 0.10; *P < 0.05;**P < 0.01;***P < 0.001 (see details and exact *P*-values in Supplementary Data 1). *n* = 150 biologically independent samples for belowground AM fungal symbionts, fungal pathogens, fungal decomposers, protistan bacterivores, protistan parasites, protistan omnivores, insect herbivores, arthropod predators and aboveground primary producers, avian herbivores; *n* = 149 biologically independent samples for aboveground vertebrate predators; *n* = 148 biologically independent samples for belowground bacterial decomposers; *n* = 144 biologically independent samples for aboveground fungal pathogens; *n* = 139 biologically independent samples for belowground arthropod decomposers and aboveground insect herbivores, arthropod omnivores, arthropod predators; *n* = 134 biologically independent samples for aboveground molluscan herbivores, molluscan omnivores; *n* = 113 biologically independent samples for aboveground insect pollinators.

General patterns regarding the drivers that structure above- and belowground communities could be observed (Fig. 3 and Supplementary Data 1). Increased plot-level land-use intensity reduced the species richness of seven of the ten aboveground groups (*P* < 0.001 for primary producers, fungal pathogens, insect herbivores and vertebrate predators, *P* < 0.01 for avian herbivores and *P* < 0.05 for molluscan herbivores and molluscan omnivores), and their abundance (Supplementary Fig. 5, *P* < 0.001 for fungal pathogens and avian herbivores, and *P* < 0.05 for molluscan herbivores, insect pollinators and molluscan omnivores). In contrast, plot-level land-use intensity had neutral or even positive effects on belowground groups (positive effects with *P* < 0.001 for fungal pathogens and protistan parasites, and *P* < 0.05 for fungal decomposers) (Fig. 3, see also Supplementary Fig. 6 for the separate effects of grazing, mowing and fertilization). High land-use intensity causes direct damage to aboveground communities in grassland via frequent mowing or intensive grazing^62,63^ and decreases plant diversity, which in turn can decrease the availability of feeding niches for higher trophic level groups aboveground^64–67^. In contrast, belowground communities are less disturbed by mowing, and fertilization may boost soil resource availability via increased organic inputs to soil, thus potentially increasing the abundance of belowground groups^53,68–70^. This, in turn, can raise the capacity for soil organisms to partition resources and coexist^71^. Meanwhile, land-use intensification might indirectly induce the spread of belowground pathogens, such as fungal pathogens or protistan parasites, by reducing plant species richness and homogenizing the aboveground plant-host community, thus reducing dilution effects and allowing pathogens to flourish^63^.

### Field-level drivers

Plant diversity at the surrounding field level positively affected the diversity of aboveground groups, but was not a strong driver of belowground trophic groups (Fig. 3). A high plant community heterogeneity (i.e. spatial species turnover, see Methods) at the field level promoted the diversity of three mobile groups: insect pollinators (*P* < 0.01), avian herbivores (*P* < 0.05) and vertebrate predators (*P* < 0.05) (Fig. 3). In agricultural environments, mobile species often exploit a wide variety of habitats in their daily lives to meet their nesting and feeding requirements, and plant community heterogeneity at the field- and landscape level may represent this variety both directly, as range of feeding opportunities, and as a proxy of habitat complexity^72^. In contrast, the lack of response of belowground groups to this factor is likely tied to their low movement abilities; most of the belowground taxa studied are sessile or immobile at the scales considered, and so are likely unaffected by field-level factors in their daily lives.

### Landscape-level drivers

**Fig. 4:**
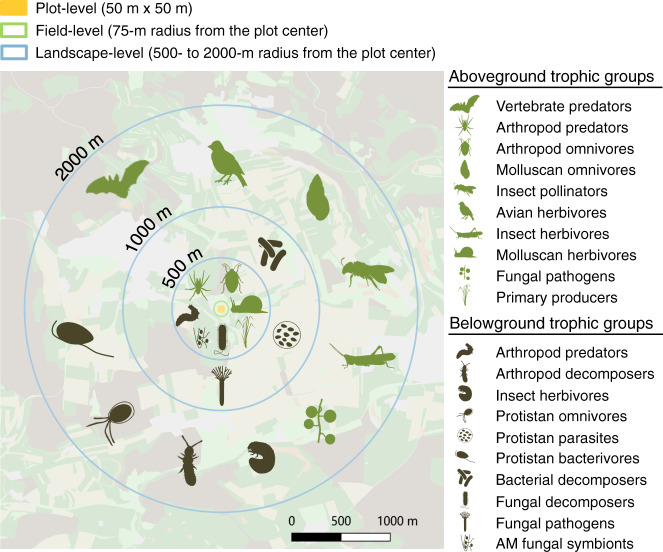
Spatial scales of landscape land-use influence on the species richness of multiple above- and belowground trophic groups. Icons within each radius show the groups whose species richness was best explained by the respective spatial scale, identified using the second-order Akaike information criterion (AICc). The scale leading to the lowest AICc in model selection was retained (see also Supplementary Table 2).

Landscape-level land use was the strongest driver of both above- and belowground biodiversity (Fig. 2), with all above- and belowground groups responding to at least one landscape-level land-use factor. We found that six of the ten aboveground trophic groups (fungal pathogens, insect herbivores, avian herbivores, insect pollinators, molluscan omnivores and vertebrate predators) were affected by the wider landscape (2000-m radius), while the responses of primary producers, molluscan herbivores, arthropod omnivores and arthropod predators were best explained by smaller-scale landscape land use (500-m radius) (Fig. 4). Notably, seven of the ten belowground groups (fungal pathogens, bacterial decomposers, protistan bacterivores, protistan parasites, protistan omnivores, insect herbivores and arthropod decomposers) were also affected by the wider landscape (1000- or 2000-m radii), while the other three (AM fungal symbionts, fungal decomposers and arthropod predators) were affected by their immediate surroundings (500-m radius) (Fig. 4).

Although above- and belowground communities responded to landscape land use at similar scales, the identity and impact of the landscape factors that shaped their diversity differed. Most of the belowground trophic groups responded strongly and positively to the presence and permanency of forests and grasslands in the surrounding landscape (Fig. 3). The species richness of five of the ten belowground trophic groups was higher in plots surrounded by high forest cover (*P* < 0.001 for bacterial decomposers, *P* < 0.01 for protistan omnivores, *P* < 0.05 for protistan bacterivores and protistan parasites and *P* < 0.10 for fungal decomposers). In addition, a high proportion of grassland in the surrounding landscape increased the diversity of fungal pathogens (*P* < 0.10), bacterial decomposers (*P* < 0.05) and protistan parasites (*P* < 0.05). Landscape-level land-use history was also an important driver of belowground biodiversity, with greater species richness of fungal pathogens (*P* < 0.05) and fungal decomposers (*P* < 0.05) in plots surrounded by permanent forest cover. Conversely, in landscapes where grassland cover was permanent there was a greater species richness of AM fungal symbionts (*P* < 0.05), fungal pathogens (*P* < 0.10), fungal decomposers (*P* < 0.05), bacterial decomposers (*P* < 0.01) and arthropod predators (*P* < 0.05) (Fig. 3). The observed effects of landscape-level land use on belowground diversity are unlikely to be driven by co-varying environmental factors as we controlled for the effects of regional differences, soil pH and texture, and topography (see Supplementary Data 2 and Methods). In contrast to belowground groups, aboveground trophic groups were more strongly affected by the current diversity of land-cover types in the landscape (Fig. 3). Land-cover diversity increased the species richness of aboveground fungal pathogens (*P* < 0.01), molluscan herbivores (*P* < 0.05), avian herbivores (*P* < 0.01), insect pollinators (*P* < 0.01) and vertebrate predators (*P* < 0.10), supporting the idea that the persistence of relatively mobile aboveground trophic groups in agricultural landscapes relies heavily on the presence of heterogenous semi-open habitats^15,22,33^.

For the effect of landscape factors on belowground diversity, we hypothesize that these effects are driven by spatial biodiversity dynamics in which permanent semi-natural habitats, such as forests and grasslands, provide stable, heterogeneous and resource-rich habitats that support a high diversity of belowground organisms, and from which species can spill-over into less suitable agricultural areas^45,73^. Furthermore, there is evidence that of these stable habitats, forests support a higher belowground diversity than grasslands, potentially explaining the relatively stronger effect of forests for several belowground groups^74^. By showing that belowground diversity is linked to habitat stability and connectivity, our study provides the most comprehensive evidence yet to support the idea that dispersal processes play a key role in shaping belowground communities^35,75–78^, including those of agricultural landscapes. This idea is further supported by field inoculation studies, which show clear evidence of local dispersal limitation^79–81^, and the very slow recovery of soil biodiversity following the cessation of agricultural practices such as tillage^48^ and biocide application^82^. More broadly, these results indicate that the concept of habitat connectivity and its measurement need to be re-thought and expanded if soil biodiversity is to be considered. However, despite the evidence we provide, it is clear that the study of spatial biodiversity dynamics in belowground communities is in its infancy^78^. Many further observational and experimental studies are required before the role of dispersal in shaping belowground communities is fully understood.

### Community restructuring

**Fig. 5:**
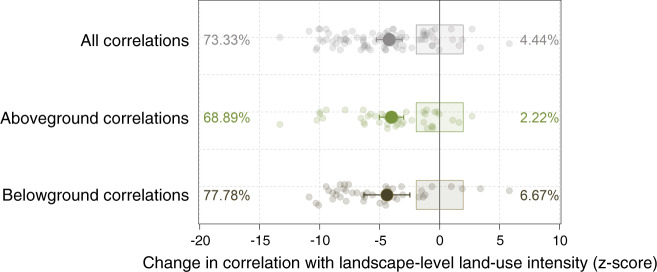
Effect of increasing landscape land-use intensity on correlations between the species richness of above- and belowground trophic groups. Z-scores (standardized effect sizes) show the changes in Pearson-correlation strength (changes in r) between the species richness of pairs of trophic groups in plots in low (*n* = 75 plots) and high (*n* = 75 plots) landscape land-use intensity. To calculate z-scores, we divided the 150 plots into 75 plots with the lowest landscape-level land-use intensity and 75 plots with the highest landscape-level land-use intensity values, and calculated the differences in Pearson coefficient of correlation. We then compared these values to a distribution of simulated r-value differences (*n* = 999) in which we randomized the values of landscape land-use intensity (low or high) between plots for each pair of trophic groups. On the basis of this random distribution, z-scores and *P*-values were calculated. Positive z*-*scores indicate increases in correlation strength between the species richness of two trophic groups at high landscape land-use intensity, and negative z*-*scores indicate decreases in correlation strengths between the species richness of two trophic groups at high landscape land-use intensity. Each coloured dot represents one correlation; larger dots represent the mean and bars the 95% confidence intervals (see details and exact *P*-values in Supplementary Data 3). Coloured rectangles separate *P-*value levels (*P* < 0.05 for dots outside the rectangle and not significant for dots inside). Percentages of positive and negative significant changes in correlation are indicated. See also Supplementary Fig. 7.

The contrasting magnitude of the response of different trophic groups to landscape-level land use indicates that the entire community may restructure under landscape land-use intensification, leading to altered relationships between trophic levels, a phenomenon that is observed for both above- and belowground taxa at the plot level in these grasslands^29,60^ (see also Supplementary Fig. 7). To provide an initial test of this hypothesis, we calculated the correlation between the species richness values of all above- and belowground trophic groups. This was done separately for plots situated in landscapes of low and high land-use intensity, using a compound measure of landscape-level land-use intensity (see Methods). We found that 73.33% of the correlations were significantly weaker in landscapes of high land-use intensity, compared to low intensity landscapes (Fig. 5). This reduction of correlation strength was stronger for correlations between belowground groups (77.78% of the correlations significantly dropped), than for those between aboveground groups (68.89% of the correlations significantly dropped), and strongly affected correlations between adjacent trophic levels (Supplementary Fig. 7, and see also Supplementary Fig. 8 for a comparison of plot-level land-use intensity). These results suggest that the loss of permanent semi-natural habitats at the landscape level disrupts interactions between specialist partners, particularly belowground, leading to large-scale community reorganisation towards a species-poor and more generalist community.

### Response of rare and common species

Finally, because the responses of common and rare species to land-use intensification and habitat simplification can differ greatly^5,14^, we investigated the response of species richness among common (i.e. those accounting for 80% of the total occurrence) and rare species (i.e. species accounting for the remaining 20% of the total occurrence) of each trophic group in separate models (see Methods). We found that the effects of permanent semi-natural habitats in the landscape tended to be more positive for rare species than for common species (Supplementary Figs. 9, 10). We hypothesize that this is due to the more particular habitat requirements of rare species, which rely strongly on strongholds of semi-natural habitats for their persistence, and which may be absent altogether from the species pool of more intensive landscapes. While the role of such refuges is well established for many rare aboveground species^7^, evidence for semi-natural habitat refuges for rare belowground species has not been presented before to our knowledge. We also found that temporal variation in plot- and field-level land use generally increases the diversity of the rarest species, a pattern that held for all trophic groups. Locally, temporal variation in land use can promote biodiversity by creating niches, allowing species with different strategies to coexist^5^. However, our results suggest that these rare species might only be able to recolonize the field if suitable habitats are present in the surrounding landscape (see also Supplementary Fig. 4).

### Conservation of above- and belowground diversity

Our comprehensive dataset allowed us to reveal how different components of land-use intensity differentially affect the above- and belowground biodiversity of multiple trophic groups. In particular, we found that local land-use intensification had opposing effects on above- and belowground diversity and that heterogeneity at field- and landscape-levels was more important for above- than belowground diversity which, in contrast, depended strongly on permanent habitats in the surrounding landscape. Although responses to the studied factors may differ for other agroecosystem types (e.g. croplands) and taxa (e.g. soil nematodes) we nevertheless present the most complete picture to date of how an entire community responds to land-use intensification. To date, conservation strategies for agroecosystems have focused on aboveground biodiversity. However, our findings support the idea that belowground biodiversity does not mirror aboveground diversity at local scales^24,26,29,83^ and suggest that a broader view is required if whole-ecosystem diversity is to be conserved.

There is now growing recognition that belowground biodiversity is a significant proportion of overall biodiversity, that it is an essential component of sustainable, productive and multifunctional agricultural landscapes, and that it requires conservation^50,84,85^. Despite this recognition, conservation actions in agroecosystems remain focused on aboveground organisms and strategies for belowground biodiversity conservation remain very simple. Our results show that an aboveground-centric conservation strategy may not be sufficient to protect belowground biodiversity. For example, a strategy for the promotion of aboveground diversity, based on our results, would limit plot-level intensification, conserve and promote diverse habitats within and in the immediate surroundings of the fields, and encourage a wider diversity of habitat types in the landscape. These actions are all commonly found as recommendations in existing agri-environment schemes^7,86^. In contrast, our results indicate that belowground biodiversity would be best promoted by an increase in plot-level grassland land-use intensity, strong restrictions on grassland tillage and conversion over long time periods, and the protection of surrounding permanent forest habitat in the landscape. Such practices tend not to be considered in agri-environment schemes that promote many activities, e.g. the sowing of flower strips, which may do little to benefit belowground diversity. Clearly, the actions that promote soil biodiversity need to be considered carefully as they may trade-off with the protection of the aboveground diversity, and further work is required to minimize these trade-offs and identify options that promote both parts of the community.

Both above- and belowground diversity play an essential role in agroecosystem function and food security^30,50,87^. Although they respond differently to the many components of land-use intensity the diversity of both was low in highly artificial and disturbed agricultural landscapes. Therefore, a broad and consistent message of our results is that there is a great need to create and protect diverse and permanent habitats within agricultural landscapes if we are to preserve both above- and belowground biodiversity and the vital services they provide.

## Methods

### Study area

The study was conducted as part of the long-term Biodiversity Exploratories project (www.biodiversity-exploratories.de) in three German regions: (i) the UNESCO Biosphere Reserve Schwäbische Alb in the low mountains of south-western Germany; (ii) the Hainich National Park and surrounding areas in hilly central Germany and (iii) the UNESCO Biosphere Reserve Schorfheide-Chorin in the post-glacial lowlands of north-eastern Germany. The three regions differ in climate, geology and topography, but each is characterized by a gradient of grassland land-use intensity that is typical for large parts of temperate Europe^49^. In each region, 50 plots (50 m × 50 m) were chosen in mesic grasslands by stratified random sampling from a total of 500 candidate plots on which initial vegetation, soil and land-use surveys were conducted. This ensured that the plots covered the whole range of land-use intensities and management types, while minimizing confounding factors such as spatial position or soil type. All plots were grasslands for at least 10 years before the start of the project in 2006, although land-use intensity varies between years^5,59^.

### Plot species richness

At each plot, we measured the species or family richness of 20 trophic groups using standard methodology (Supplementary Table 3). In total we observed taxa spanning ten aboveground and ten belowground trophic groups. The aboveground trophic groups were: primary producers (vascular plants), fungal pathogens (foliar fungal pathogens including rust, powdery mildew, downy mildew and smut fungi), molluscan herbivores (molluscs feeding on plant material), insect herbivores (insects feeding on plant material), avian herbivores (herbivorous birds), insect pollinators (insects feeding on plant pollen or nectar), molluscan omnivores (molluscs feeding on both animals and plants), arthropod omnivores (insects and harvestmen feeding on both animals and plants), arthropod predators (carnivorous insects, spiders and Chilopoda) and vertebrate predators (insectivorous birds and bats). The belowground trophic groups were: fungal symbionts (arbuscular mycorrhizal fungi), fungal pathogens (soil-borne plant fungal pathogens), fungal decomposers (soil-borne fungal decomposers), bacterial decomposers (soil-borne bacteria), protistan bacterivores (bacteria-feeding protists), protistan omnivores (consumer of other protists, fungi, and algae), protistan parasites (protists parasitising a range of organisms), insect herbivores (herbivorous insect larvae sampled underground), arthropod decomposers (arthropods feeding on litter and other detritus) and arthropod predators (carnivorous arthropods sampled underground).

We sampled vascular plants in an area of 4 m × 4 m on each plot, and estimated the percentage cover of each occurring species (see also Supplementary Table 3). Fungal pathogens were sampled in four transects of 25 m × 1 m per plot. We inspected all occurring vascular plant species for infested individuals, sampled them and later identified the pathogenic fungi to the species level. Aboveground molluscan herbivores and omnivores were sampled by collecting five surface samples per plot (20 cm × 20 cm, about 2 cm deep), using a sharp knife, along with the herbaceous vegetation, mosses, litter and the upper soil layer. For sampling aboveground insect herbivores, pollinators, arthropod omnivores and predators we used sweep netting by conducting 60 double sweeps along three 50-m plot-border transects. Additionally, some insect pollinators were hand-collected during their visits on flowers, identified and individuals counted (Diptera and Hymenoptera), or recorded within 30 min along a 300-m transect (Lepidoptera). Avian herbivores and vertebrate predators were sampled by audio-visual point counts, at the center of the respective grassland plot (50 m × 50 m) for birds and along two 200-m plot-border transects for bats. Acoustic recordings of bats were taken in real time with a Pettersson-D1000x bat detector (Pettersson Electronic AG, Uppsala, Sweden). Bat species identification was then conducted using the software Avisoft SAS Lab Pro, Version 5.0.24 and onward (Raimund Specht, Avisoft Bioacoustics, Berlin Germany). To sample belowground AM fungal symbionts, fungal pathogens, fungal decomposers, bacterial decomposers and protistan bacterivores, parasites and omnivores, fourteen soil cores (diameter 4.8 cm) were taken from a 20 m × 20 m subarea of each grassland plot, and soil from the upper 10 cm of the upper horizon was homogenized after removal of root material. The bulk sample was split into subsamples for the analyses of AM fungal symbionts, fungal pathogens, fungal decomposers, bacterial decomposers and protistan bacterivores, parasites and omnivores. Belowground insect herbivores, arthropod predators and arthropod decomposers were sampled by collecting soil cores (maximum diameter 20 cm, maximum depth 10 cm) from each plot. Soil fauna was extracted from soil cores using a modified heat extraction system over a period of eight days, while the soil macrofauna was hand-sorted. In 2019, belowground arthropod decomposers were extracted as a composite sample from nine soil cores (diameter 4.5 cm, depth 10 cm). A subsample of this composite sample was used to identify the major taxonomic groups to species level. In addition to soil extraction, some belowground arthropod decomposer species were sampled with sweep netting (60 double sweeps along three 150-m plot-border transects). While these taxa were sampled aboveground, they are known detritivores, and so classified as belowground organisms (see also Supplementary Table 3).

We directly measured species richness for most groups, but used family richness for belowground insects, the number of different amplicon sequence variants (ASV) for AM fungal symbionts, fungal pathogens, fungal decomposers, bacterial decomposers, and the number of different operational taxonomic units (OTU) for protists. For AM fungal symbionts, fungal pathogens and fungal decomposers, DNA was extracted using ‘MO BIO Power Soil DNA isolation kit’ (MO BIO Laboratories, Carlsbad, CA, USA) following the manufacturer’s protocol. We then use a Illumina Hiseq platform for the sequencing, and sequence reads were processed using plugins available in the QIIME 2™ plattform (https://qiime2.org/, Version 2017.12). For bacterial decomposers, RNA was extracted using a custom protocol (Lueders protocol). For protists, soil DNA was extracted using the DNeasy PowerSoil Kit (Qiagen GmbH, Hilden, Germany) (see also Supplementary Table 3).

Additionally, we calculated species richness for common and rare species separately. For each species (or family for belowground insects, ASV for bacterial decomposer, AM fungal symbionts, fungal pathogens, fungal decomposers and OTU for protists), we calculated its total occurrence across all plots. Within each of the 20 trophic groups, we split the species into two categories: ‘common’ species were those accounting for 80% of the total occurrence, while the other species were considered ‘rare’.

### Environmental factors

In each grassland plot, we measured hypothetically important environmental covariates, related to potential drivers of species richness (Supplementary Table 1). Soil depth was measured as the combined thickness of all topsoil and subsoil horizons. We determined soil depth by sampling a soil core in the center of the study plots. We used a motor driven soil column cylinder with a diameter of 8.3 cm for the soil sampling (Eijkelkamp, Giesbeek, The Netherlands). For the other soil parameters, a composite sample representing the soil of the whole plot was prepared by mixing 14 mineral topsoil samples (0–10 cm, using a manual soil corer with 5.3 cm diameter) from the same plot. Soil samples were air dried and sieved (<2 mm), and we then measured the soil pH in the supernatant of a 1:2.5 mixture of soil and 0.01 M CaCl_2_. Finally, for each plot we calculated Topographic Wetness Index (TWI), defined as ln(*a*/tan*B*) where *a* is the specific catchment area (cumulative upslope area which drains through a Digital Elevation Model (DEM, http://www.bkg.bund.de) cell, divided by per unit contour length) and tan*B* is the slope gradient in radians calculated over a local region surrounding the cell of interest^88,89^. TWI therefore combines both upslope contributing area (determining the amount of water received from upslope areas) and slope (determining the loss of water from the site to downslope areas). TWI was calculated from raster DEM data with a cell size of 25 m for all plots, using GIS tools (flow direction and flow accumulation tools of the hydrology toolset and raster calculator). The TWI measure used was the average value for a 4 × 4 window centred on the plot, i.e. 16 DEM cells corresponding to an area of 100 m × 100 m. Initial analyses found that this was a stronger predictor than more local measures, thus indicating it is representative of the 50 m × 50 m plot area and its surroundings.

### Plot land use

At the plot level (50 m × 50 m, Fig. 1), land use was assessed annually via questionnaires sent to land managers in which they reported the level of fertilization (kg N ha^−1^ yr^−1^), the number of mowing events per year (from one to three cuts), and the number and type of livestock and their duration of grazing (number of livestock units ha^−1^ yr^−1^). We used this information to calculate three standardized indices summarizing grazing, fertilization and mowing intensity^51,59^. Each component was divided by the global mean value across all three regions and across all years to standardize the components. Within the grassland fields considered in this study, mowing and fertilization intensities are positively correlated (r = 0.70), while grazing and mowing intensities are negatively correlated (r = −0.61) (Supplementary Data 2). Due to these correlations, independent effects of each land-use component cannot be reliably estimated. We therefore used a compound index of plot land-use intensity. The land-use intensity index (LUI) was calculated as the global mean of grassland management across the three regions overall the years of 2006–2017 according to^51^, using the LUI calculation tool^90^ implemented in BExIS (10.17616/R32P9Q). We calculated the mean LUI for each plot over the years 2006–2017 because this reflects the average LUI around the years when most of the data was collected. At the minimum LUI of 0.5–0.7, grasslands are typically unfertilized, not mown, and grazed by one cow (>2 years old) per hectare for 30 days (or one sheep per hectare for the whole year). At an intermediate LUI of 1.5, grasslands are usually unfertilized (or fertilized with less than 30 kg N ha^−1^y^−1^), and are either mown twice a year or grazed by one cow per hectare for most of the year (300 days). At a high LUI of 3, grasslands are typically fertilized at a rate of 60–120 kg N ha^−1^y^−1^, are mown 2–3 times a year or grazed by three cows per hectare for most of the year (300 days), or are managed by a combination of grazing and mowing. Very high intensity grasslands (e.g. those cut more than three times per year and ploughed annually) are not found within the study regions. We also quantified the interannual variation of land-use intensity calculated as the standard deviation (sd) in LUI (hereafter called ‘variation in land-use intensity’) as this is known to be related to the species diversity of several aboveground trophic groups^5^. As biodiversity has been shown to have exponential responses to LUI and LUI sd, we log-transformed these two predictors and the response variables^5,29^. Note that the variation in land-use intensity was not highly correlated with the mean LUI across years (r = 0.40) (Supplementary Data 2).

### Field plant diversity and land use

To assess the surrounding plant diversity of each grassland plot, we surveyed the vegetation within the major surrounding homogeneous vegetation zones in a 75-m radius of each plot (i.e. field level, Fig. 1) in 2017 and 2018. These zones were mostly situated within the same grassland field as the focal plot but we occasionally surveyed other habitat types (c. 20% were situated in hedgerows, margins or forests). In each of these zones, we selected a single, representative area of 2 m × 2 m in which the cover of all vascular plant species was estimated. We surveyed at least four quadrats for each grassland plot. To do so, if less than four different homogeneous zones were identified, we surveyed the vegetation twice or more within a homogeneous zone. We then calculated the changes in species composition between these surrounding plant communities (hereafter called ‘plant community heterogeneity’) as the Sørensen dissimilarity index^91^. Field plant community heterogeneity was used to represent habitat and resource diversity in the immediate surroundings of the plot for above- and belowground trophic groups, as a high plant species turnover is closely related to environmental heterogeneity and the beta diversity of microbes^92,93^. Additionally, we used historical land-use maps to calculate the temporal variation in field land use. Historical maps from the Schwäbische Alb are digitized cadastral maps from 1820, topographic maps (map scale = 1:25,000) from the German Empire from 1910, and topographic maps (map scale = 1:25,000) from the Federal Republic of Germany from 1960. Historical maps from the Hainich are digitized old topographic maps (map scale = 1:25,000) from 1850, topographic maps (map scale = 1:25,000) from the German Empire from 1930, and topographic maps (map scale = 1:10,000) from the German Democratic Republic from 1960. Historical maps from Schorfheide-Chorin are digitized old topographic maps (map scale = 1:25,000) of 1850, topographic maps (map scale = 1:25,000) from the German Empire of 1930, and topographic maps (map scale = 1:25,000) from the German Democratic Republic from 1960. Variation in field land use varied between 0 (the field was always recorded as a grassland since 1820/50) and 3 (the land use recorded at the field level was different between 1820/50 and 1910/30, and between 1910/30 and 1960).

### Landscape land use

At the landscape level (i.e. up to 2000 m of the center of each grassland plot, Fig. 1), land use was recorded in 2008 within a 2000-m radius of each grassland plot, and mapped in a Geographical Information System (GIS) database, running on QGIS v 3.6. Land use was classified into five broad categories: croplands, grasslands, forests, water bodies, roads and urban areas (Supplementary Table 4). To describe the current landscape-level land use, we calculated the proportion of the landscape covered by grasslands and forests. Forests represent less disturbed habitats in agricultural landscapes and are likely to act as favourable habitats and dispersal corridors for some of the taxa studied^45,94^. We also calculated the diversity of land-cover types in the landscape (i.e. the Shannon diversity of land-cover types), which has been shown to positively affect biodiversity in agricultural landscapes^14,33,95^. A second landscape land-use survey was done in 2017 and we found that the grassland cover (r = 0.81), the forest cover (r = 0.80) and the total land-cover diversity (r = 0.71) recorded in 2017 were highly correlated with data using a 250-m radius of each grassland plot in 2008, suggesting that over the last 10 years landscape land use is largely unchanged. Additionally, we used the historical land-use maps to quantify the permanency of the forest and grassland covers between 1820/50 and 2008. To do so, we calculated the ratio of the mean forest or grassland cover recorded in the landscape from 1820/50 to 2008, to the standard deviation of the forest or grassland cover over that period. Forest or grassland permanency values were high when there was a high forest or grassland cover over time and this cover did not fluctuate. Landscape permanency differs from temporal variation in field land use in that a grassland field may have been a permanent habitat for many years while the surrounding landscape units saw significant changes, and vice versa. This difference is reflected by the weak correlation between these variables (−0.45 < r < 0.13). Both current and historical landscape composition predictors were calculated in a 500-m radius, 1000-m radius and 2000-m radius of the center of each grassland plot. As biodiversity can have non-linear response to landscape predictors^7^, landscape land-use predictors were also log-transformed.

In addition to the plot-level LUI index, we calculated a landscape land-use intensity index by calculating the inverse of the sum of the standardised values of land-cover diversity, forest and grassland cover, and forest and grassland cover permanency at the 1000-m scale. These variables had approximately equal importance in driving the species richness of above- and belowground trophic groups (see Fig. 2). Landscape land-use intensity was considered high when the landscape had low land-cover diversity, forest and grassland cover, and forest and grassland cover permanency (i.e. high values of landscape land-use intensity index). At the 1000-m scale, the landscape land-use intensity index was positively correlated with the annual crop cover, a metric associated with landscape land-use intensity^14^ (Supplementary Data 2).

### Data analysis

All analyses were performed using R version 4.0.3^96^. To assess the role of multiple environmental factors, and plot-, field- and landscape-level factors (standardized to give comparable coefficients) in driving the species richness of each of the ten above- and ten belowground trophic groups, we fitted linear models (using the lm function). Both within and across the three regions of our study many environmental factors, e.g. climate, soil type and elevation are strongly confounded, making independent assessment of these environmental factors difficult to estimate. Therefore, to account for inherent regional differences in environmental conditions and to focus on the independent effects of the focal plot-, field- and landscape-level factors, we first fitted the term ‘region’ as a predictor in a linear model and calculated residuals for all our variables (both explanatory and response variables), and then used these residual values in all subsequent analyses. As an alternative to using residuals, we also fitted models with the region term as predictor, as well as the environmental factors, plot-, field- and landscape-level factors (standardized to give comparable coefficients) alongside the diversity measures. This approach gave very similar results (Supplementary Figs. 1, 2).

We considered four groups of predictors, spanning a range of spatial scales, in our linear model containing the following terms: (i) environmental factors: soil pH, soil clay content, and the TWI; (ii) plot-level land use, represented by two terms, LUI and interannual variation in LUI; (iii) the field level (75-m radius of the plot) plant diversity and land use, represented by two terms: plant community heterogeneity and variation in field land use; (iv) the landscape-level land use, represented by five terms: land-cover diversity, forest cover, grassland cover, forest cover permanency and grassland cover permanency (i-iii are considered local drivers, while *iv* landscape drivers—see introduction). In the early stages of our analyses, we considered the use of far more variables to describe each group of predictors (i.e. a wide range of environmental factors, plot-level factors, field-level factors and landscape-level factors). However, in order to make our manuscript comprehensible and to allow comparison between responses of above- and belowground groups, we choose to focus on a relevant, standardized and simplified subset of variables that are hypothesized to be strong drivers of above- and belowground biodiversity (see Supplementary Table 1). In addition, due to strong correlations between some of the variables considered, multicollinearity would have likely been an issue in our models. Since soil sand content (r = −0.75) and soil depth were highly correlated (r = −0.72) with soil clay content (Supplementary Data 2), we chose to use only soil clay content to represent soil texture in our analyses, as it has been shown to be a strong driver of belowground biodiversity^53^. To ensure normal error distributions and homogenous variance, we log-transformed all response variables. After this transformation, we visually checked for non-linear relationships in preliminary analysis but did not find any evidence of such relationships and so fitted only linear terms in our final models. In all cases, we used a Gaussian error distribution as the errors of our response variables were normally distributed. All predictors and response variables were scaled (z-scored) to allow comparison of effects and responses between trophic groups.

For each trophic group, we fitted three competing models in which the landscape land-use factors were calculated either in a 500-m radius, 1000-m radius or 2000-m radius of the grassland plot. We then selected the model for which the second-order Akaike information criterion (AICc) was lowest. When the AICc of the models were separated by a Δ AICc < 2, we retained the model with the largest spatial scale^97^ (Supplementary Table 2). To assess the sensitivity of our results to this approach we also used a fixed 1000-m radius for landscape effects for all trophic groups, a commonly used scale when investigating the effects of the landscape context on aboveground biodiversity^7,33,34^. We found that the direction and magnitude of effects of the different factors on the species richness of multiple above- and belowground trophic groups were comparable to those estimated by the variable radius models when using this fixed term (Supplementary Fig. 11). Because the radii of landscape factors overlap between neighbouring sites at larger spatial scales, potentially leading to issues of non-independence, we re-ran these analyses to consider only a subset of sites with non-overlapping landscape radii. Considering only this subset of plots did not affect our estimates of the importance of landscape factors in driving the species richness of the different trophic groups (Supplementary Fig. 3).

We report the effects of environmental factors, plot-, field- and landscape-level factors on the different trophic groups as slopes from the linear model. Model residuals were inspected for constant variance and normality, which found that assumptions of homoscedasticity were met. We tested for residual spatial autocorrelation using Moran’s I tests and did not find any evidence of residual spatial autocorrelation (*P*-values > 0.10). Correlation among the predictors used in the models (−0.60 < r < 0.75) (Supplementary Data 2) did not induce multicollinearity issues in our analyses (Supplementary Table 5). To reduce potential type I errors associated with multiple testing while minimizing type II errors, we controlled for false discovery rates (FDR) using a Benjamini–Hochberg procedure^98^ with a threshold of 0.2^99^.

To evaluate the relative importance of (i) environmental factors, (ii) plot-level land use, (iii) field-level plant diversity and land use and (iv) landscape-level land use as drivers of above- and belowground biodiversity, we expressed the importance of each group of predictors as the percentage of variance they explained, based on the comparison between the absolute values of their standardized regression coefficients and the sum of all standardized regression coefficients from the model. This method is similar to a variance partitioning analysis because we previously transformed all predictors to z-scores^33,100,101^. Additionally, in separate models we analysed first order interactive effects between the landscape land use, and the plot or field land use, as land use in the landscape may modulate the effects of current plot and field land use on biodiversity. As interactive effects were never major drivers of biodiversity, we present only main effects here, but see Supplementary Fig. 4 for details of interactive effects between the drivers.

In follow up analyses, we accounted for the effects of the three land-use components (fertilization, grazing and mowing) separately, by running the same series of models but replacing the LUI by the individual land-use components. In addition, we ran the same models on the biomass (primary producers) or abundance (all other groups) of all aboveground trophic groups, and on the abundance of belowground insect herbivores, arthropod decomposers and arthropod predators. We did not have data on the abundance of the phylotypes of AM fungal symbionts, fungal pathogens, fungal decomposers, bacterial decomposers, protistan bacterivores, protistan omnivores and protistan parasites, making a proper comparison of land-use effects on the abundance of above- and belowground groups impossible. We also analysed the responses of common and rare species by using the same series of models but on the species richness of common and rare species subsets of each trophic group.

Finally, to test for possible effects of landscape-level land use on the correlations between the diversities of the trophic groups, we calculated the observed correlation between all pairs of above- and belowground trophic groups. This was performed separately for plots situated in landscapes with low land-use intensity (low landscape land-use intensity index, i.e. landscapes with high land-cover diversity, forest and grassland covers and forest and grassland permanency) and those in high land-use intensity landscapes (high landscape land-use intensity index, i.e. landscapes with low land-cover diversity, forest and grassland covers, and forest and grassland permanency). To do this we divided the 150 plots into 75 plots with the lowest landscape-level land-use intensity and 75 plots with the highest landscape-level land-use intensity values, and calculated the differences in Pearson coefficient of correlation (r). We then compared these values to a distribution of simulated r-value differences (*n* = 999) in which we randomized the values of landscape land-use intensity (low or high) between plots for each pair of trophic groups. On the basis of this random distribution, we calculated z-scores (standardized effect sizes) and *P*-values. Significant values thus indicate stronger trophic interactions in grasslands surrounded by a landscape with lower (or higher) land-use intensity than expected by chance. We ran a similar analysis considering the 75 plots with the lowest plot-level land-use intensity and the 75 plots with the highest plot-level land-use intensity.

### Reporting summary

Further information on research design is available in the Nature Research Reporting Summary linked to this article.

## Supplementary information

Supplementary Information

Description of Additional Supplementary Files

Supplementary Data 1

Supplementary Data 2

Supplementary Data 3

Reporting Summary

## Data Availability

This work is based on data from several projects of the Biodiversity Exploratories programme (DFG Priority Program 1374). The data used for analyses are publicly available from the Biodiversity Exploratories Information System (10.17616/R32P9Q), or will become publicly available after an embargo period of 5 years from the end of data assembly to give the owners and collectors of the data time to perform their analysis. Any other relevant data are available from the corresponding author upon reasonable request.

## References

[1] Kleijn, D. et al. On the relationship between farmland biodiversity and land-use intensity in Europe. *Proc. R. Soc. Lond. B Biol. Sci*. **276**, 903–909 (2009). 10.1098/rspb.2008.1509 PMC2664376 19019785

[2] Ollerton J, Erenler H, Edwards M, Crockett R (2014). Extinctions of aculeate pollinators in Britain and the role of large-scale agricultural changes. Science.

[3] Stanton RL, Morrissey CA, Clark RG (2018). Analysis of trends and agricultural drivers of farmland bird declines in North America: a review. Agric. Ecosyst. Environ..

[4] Beckmann M (2019). Conventional land-use intensification reduces species richness and increases production: a global meta-analysis. Glob. Change Biol..

[5] Allan E (2014). Interannual variation in land-use intensity enhances grassland multidiversity. Proc. Natl Acad. Sci. USA.

[6] Newbold, T. et al. Has land use pushed terrestrial biodiversity beyond the planetary boundary? A global assessment. *Science* **353**, 288–291 (2016). 10.1126/science.aaf2201 27418509

[7] Le Provost G (2020). Land-use history impacts functional diversity across multiple trophic groups. Proc. Natl Acad. Sci. USA.

[8] Geiger F (2010). Persistent negative effects of pesticides on biodiversity and biological control potential on European farmland. Basic Appl. Ecol..

[9] Rajaniemi TK (2002). Why does fertilization reduce plant species diversity? Testing three competition-based hypotheses. J. Ecol..

[10] Zeng J (2016). Nitrogen fertilization directly affects soil bacterial diversity and indirectly affects bacterial community composition. Soil Biol. Biochem..

[11] Suding KN (2005). Functional-and abundance-based mechanisms explain diversity loss due to N fertilization. Proc. Natl Acad. Sci. USA.

[12] Perović D (2015). Configurational landscape heterogeneity shapes functional community composition of grassland butterflies. J. Appl. Ecol..

[13] Redlich S, Martin EA, Wende B, Steffan-Dewenter I (2018). Landscape heterogeneity rather than crop diversity mediates bird diversity in agricultural landscapes. PLoS ONE.

[14] Gámez-Virués S (2015). Landscape simplification filters species traits and drives biotic homogenization. Nat. Commun..

[15] Benton TG, Vickery JA, Wilson JD (2003). Farmland biodiversity: is habitat heterogeneity the key?. Trends Ecol. Evol..

[16] Gonthier DJ (2014). Biodiversity conservation in agriculture requires a multi-scale approach. Proc. R. Soc. Lond. B Biol. Sci..

[17] Leibold MA (2004). The metacommunity concept: a framework for multi-scale community ecology. Ecol. Lett..

[18] Chase JM, Myers JA (2011). Disentangling the importance of ecological niches from stochastic processes across scales. Philos. Trans. R. Soc. Lond. B Biol. Sci..

[19] Thompson PL (2020). A process-based metacommunity framework linking local and regional scale community ecology. Ecol. Lett..

[20] Gravel D, Canham CD, Beaudet M, Messier C (2006). Reconciling niche and neutrality: the continuum hypothesis. Ecol. Lett..

[21] Vellend M (2010). Conceptual synthesis in community ecology. Q. Rev. Biol..

[22] Tscharntke T, Klein AM, Kruess A, Steffan-Dewenter I, Thies C (2005). Landscape perspectives on agricultural intensification and biodiversity–ecosystem service management. Ecol. Lett..

[23] Blitzer EJ (2012). Spillover of functionally important organisms between managed and natural habitats. Agric. Ecosyst. Environ..

[24] Birkhofer K (2017). Land-use type and intensity differentially filter traits in above- and below-ground arthropod communities. J. Anim. Ecol..

[25] de Graaff M-A, Hornslein N, Throop HL, Kardol P, van Diepen LTA (2019). Effects of agricultural intensification on soil biodiversity and implications for ecosystem functioning: a meta-analysis. Adv. Agron..

[26] De Deyn GB, Van der Putten WH (2005). Linking aboveground and belowground diversity. Trends Ecol. Evol..

[27] Field R (2009). Spatial species-richness gradients across scales: a meta-analysis. J. Biogeogr..

[28] Cameron EK (2019). Global mismatches in aboveground and belowground biodiversity. Conserv. Biol..

[29] Gossner MM (2016). Land-use intensification causes multitrophic homogenization of grassland communities. Nature.

[30] Geisen S, Wall DH, van der Putten WH (2019). Challenges and opportunities for soil biodiversity in the anthropocene. Curr. Biol..

[31] Tsiafouli MA (2015). Intensive agriculture reduces soil biodiversity across Europe. Glob. Change Biol..

[32] George PBL (2019). Divergent national-scale trends of microbial and animal biodiversity revealed across diverse temperate soil ecosystems. Nat. Commun..

[33] Sirami C (2019). Increasing crop heterogeneity enhances multitrophic diversity across agricultural regions. Proc. Natl Acad. Sci. USA.

[34] Seibold S (2019). Arthropod decline in grasslands and forests is associated with landscape-level drivers. Nature.

[35] Dauber J (2005). Local vs. landscape controls on diversity: a test using surface-dwelling soil macroinvertebrates of differing mobility. Glob. Ecol. Biogeogr..

[36] Cadotte MW, Fukami T (2005). Dispersal, spatial scale, and species diversity in a hierarchically structured experimental landscape. Ecol. Lett..

[37] Grilli G (2017). Fungal diversity at fragmented landscapes: synthesis and future perspectives. Curr. Opin. Microbiol..

[38] Fenchel TOM, Finlay BJ (2004). The ubiquity of small species: patterns of local and global diversity. Bioscience.

[39] Postma-Blaauw MB, Goede RGM, de, Bloem J, Faber JH, Brussaard L (2010). Soil biota community structure and abundance under agricultural intensification and extensification. Ecology.

[40] Boeraeve M, Honnay O, Jacquemyn H (2019). Local abiotic conditions are more important than landscape context for structuring arbuscular mycorrhizal fungal communities in the roots of a forest herb. Oecologia.

[41] Meyer A (2013). Different land use intensities in grassland ecosystems drive ecology of microbial communities involved in nitrogen turnover in soil. PLoS ONE.

[42] Thomson BC (2015). Soil conditions and land use intensification effects on soil microbial communities across a range of European field sites. Soil Biol. Biochem..

[43] Fahrig L (2003). Effects of habitat fragmentation on biodiversity. Annu. Rev. Ecol. Evol. Syst..

[44] Chaudhary VB, Nolimal S, Sosa-Hernández MA, Egan C, Kastens J (2020). Trait-based aerial dispersal of arbuscular mycorrhizal fungi. N. Phytol..

[45] Vannette RL, Leopold DR, Fukami T (2016). Forest area and connectivity influence root-associated fungal communities in a fragmented landscape. Ecology.

[46] Purschke O (2014). Interactive effects of landscape history and current management on dispersal trait diversity in grassland plant communities. J. Ecol..

[47] Thiel N (2020). Airborne bacterial emission fluxes from manure-fertilized agricultural soil. Microb. Biotechnol..

[48] Adl SM, Coleman DC, Read F (2006). Slow recovery of soil biodiversity in sandy loam soils of Georgia after 25 years of no-tillage management. Agric. Ecosyst. Environ..

[49] Fischer M (2010). Implementing large-scale and long-term functional biodiversity research: The Biodiversity Exploratories. Basic Appl. Ecol..

[50] Soliveres S (2016). Biodiversity at multiple trophic levels is needed for ecosystem multifunctionality. Nature.

[51] Blüthgen N (2012). A quantitative index of land-use intensity in grasslands: integrating mowing, grazing and fertilization. Basic Appl. Ecol..

[52] Kéfi S (2012). More than a meal… integrating non-feeding interactions into food webs. Ecol. Lett..

[53] Birkhofer K (2012). General relationships between abiotic soil properties and soil biota across spatial scales and different land-use types. PLoS ONE.

[54] Xue P-P, Carrillo Y, Pino V, Minasny B, McBratney AB (2018). Soil properties drive microbial community structure in a large scale transect in South Eastern Australia. Sci. Rep..

[55] Löbel S, Dengler J, Hobohm C (2006). Species richness of vascular plants, bryophytes and lichens in dry grasslands: The effects of environment, landscape structure and competition. Folia Geobot..

[56] Myers MC, Mason JT, Hoksch BJ, Cambardella CA, Pfrimmer JD (2015). Birds and butterflies respond to soil-induced habitat heterogeneity in experimental plantings of tallgrass prairie species managed as agroenergy crops in Iowa, USA. J. Appl. Ecol..

[57] Moeslund JE (2013). Topographically controlled soil moisture drives plant diversity patterns within grasslands. Biodivers. Conserv..

[58] Ågren AM, Lidberg W, Strömgren M, Ogilvie J, Arp PA (2014). Evaluating digital terrain indices for soil wetness mapping–a Swedish case study. Hydrol. Earth Syst. Sci..

[59] Vogt J (2019). Eleven years’ data of grassland management in Germany. Biodivers. Data J..

[60] Manning P (2015). Grassland management intensification weakens the associations among the diversities of multiple plant and animal taxa. Ecology.

[61] Loreau M, Mouquet N, Gonzalez A (2003). Biodiversity as spatial insurance in heterogeneous landscapes. Proc. Natl Acad. Sci. USA.

[62] Morris MG (2000). The effects of structure and its dynamics on the ecology and conservation of arthropods in British grasslands. Biol. Conserv..

[63] Socher SA (2012). Direct and productivity-mediated indirect effects of fertilization, mowing and grazing on grassland species richness. J. Ecol..

[64] Simons NK (2014). Resource-mediated indirect effects of grassland management on arthropod diversity. PLoS ONE.

[65] Harpole WS (2016). Addition of multiple limiting resources reduces grassland diversity. Nature.

[66] Pöyry J (2006). Different responses of plants and herbivore insects to a gradient of vegetation height: an indicator of the vertebrate grazing intensity and successional age. Oikos.

[67] Uchida K, Ushimaru A (2014). Biodiversity declines due to abandonment and intensification of agricultural lands: patterns and mechanisms. Ecol. Monogr..

[68] Shange RS, Ankumah RO, Ibekwe AM, Zabawa R, Dowd SE (2012). Distinct soil bacterial communities revealed under a diversely managed agroecosystem. PLoS ONE.

[69] Poulsen PHB (2013). Effects of fertilization with urban and agricultural organic wastes in a field trial—Prokaryotic diversity investigated by pyrosequencing. Soil Biol. Biochem..

[70] Filazzola A (2020). The effects of livestock grazing on biodiversity are multi-trophic: a meta-analysis. Ecol. Lett..

[71] Hooper DU (2000). Interactions between aboveground and belowground biodiversity in terrestrial ecosystems: patterns, mechanisms, and feedbacks. Bioscience.

[72] López-Jamar J, Casas F, Díaz M, Morales MB (2011). Local differences in habitat selection by Great Bustards Otis tarda in changing agricultural landscapes: implications for farmland bird conservation. Bird Conserv. Int..

[73] Boeraeve M (2018). The impact of spatial isolation and local habitat conditions on colonization of recent forest stands by ectomycorrhizal fungi. Forest Ecol. Manag..

[74] Fiore-Donno AM, Richter-Heitmann T, Bonkowski M (2020). Contrasting responses of protistan plant parasites and phagotrophs to ecosystems, land management and soil properties. Front. Microbiol..

[75] Diekötter T, Wamser S, Wolters V, Birkhofer K (2010). Landscape and management effects on structure and function of soil arthropod communities in winter wheat. Agric. Ecosyst. Environ..

[76] Decaëns T (2010). Macroecological patterns in soil communities. Glob. Ecol. Biogeogr..

[77] Hanson CA, Fuhrman JA, Horner-Devine MC, Martiny JBH (2012). Beyond biogeographic patterns: processes shaping the microbial landscape. Nat. Rev. Microbiol..

[78] Thakur MP (2020). Towards an integrative understanding of soil biodiversity. Biol. Rev..

[79] Peay K, Garbelotto M, Bruns T (2010). Evidence of dispersal limitation in soil microorganisms: isolation reduces species richness on mycorrhizal tree islands. Ecology.

[80] van der Putten WH (2012). Climate change, aboveground-belowground interactions, and species’ range shifts. Annu. Rev. Ecol. Evol. Syst..

[81] Wubs ERJ, Putten WH, van der, Bosch M, Bezemer TM (2016). Soil inoculation steers restoration of terrestrial ecosystems. Nat. Plants.

[82] Bünemann EK, Schwenke GD, Van Zwieten L (2006). Impact of agricultural inputs on soil organisms—a review. Soil Res..

[83] Cameron EK (2019). Global mismatches in aboveground and belowground biodiversity. Conserv. Biol..

[84] Guerra CA (2021). Tracking, targeting, and conserving soil biodiversity. Science.

[85] Guerra CA (2020). Blind spots in global soil biodiversity and ecosystem function research. Nat. Commun..

[86] Kleijn D, Sutherland WJ (2003). How effective are European agri-environment schemes in conserving and promoting biodiversity?. J. Appl. Ecol..

[87] Bender SF, Wagg C, van der Heijden MG (2016). An underground revolution: biodiversity and soil ecological engineering for agricultural sustainability. Trends Ecol. Evol..

[88] Gessler PE, Moore ID, McKenzie NJ, Ryan PJ (1995). Soil-landscape modelling and spatial prediction of soil attributes. Int. J. Geogr. Inf. Syst.

[89] Sørensen R, Zinko U, Seibert J (2006). On the calculation of the topographic wetness index: evaluation of different methods based on field observations. Hydrol. Earth Syst. Sci..

[90] Ostrowski, A., Lorenzen, K., Petzold, E. & Schindler, S. *Land use intensity index (LUI) calculation tool of the Biodiversity Exploratories project for grassland survey data from three different regions in Germany since 2006, BEXIS 2 module*. (Zenodo, 2020).

[91] Koleff P, Gaston KJ, Lennon JJ (2003). Measuring beta diversity for presence–absence data. J. Anim. Ecol..

[92] Prober SM (2015). Plant diversity predicts beta but not alpha diversity of soil microbes across grasslands worldwide. Ecol. Lett..

[93] Ulrich W (2014). Climate and soil attributes determine plant species turnover in global drylands. J. Biogeogr..

[94] Shoffner A, Wilson AM, Tang W, Gagné SA (2018). The relative effects of forest amount, forest configuration, and urban matrix quality on forest breeding birds. Sci. Rep..

[95] Fahrig L (2011). Functional landscape heterogeneity and animal biodiversity in agricultural landscapes. Ecol. Lett..

[96] R Core Team. *R: A language and environment for statistical computing*. (R Foundation for Statistical Computing, 2020).

[97] Ricci B (2009). The influence of landscape on insect pest dynamics: a case study in southeastern France. Landsc. Ecol..

[98] Benjamini Y, Hochberg Y (1995). Controlling the false discovery rate: a practical and powerful approach to multiple testing. J. R. Stat. Soc. Ser. B Methodol..

[99] Verhoeven KJF, Simonsen KL, McIntyre LM (2005). Implementing false discovery rate control: increasing your power. Oikos.

[100] Gross N (2017). Functional trait diversity maximizes ecosystem multifunctionality. Nat. Ecol. Evol..

[101] Le Bagousse-Pinguet Y (2019). Phylogenetic, functional, and taxonomic richness have both positive and negative effects on ecosystem multifunctionality. Proc. Natl Acad. Sci. USA.

