# Supplementary material for: Contrasting responses of above- and belowground diversity to multiple components of land-use intensity
Source: Nat Commun. 2021 Jun 24;12:3918. doi: 10.1038/s41467-021-23931-1 (PMC8225671; doi:10.1038/s41467-021-23931-1)
Supplement: Supplementary file 1 — Supplementary Information [file 41467_2021_23931_MOESM1_ESM.pdf]

Supplementary Information File

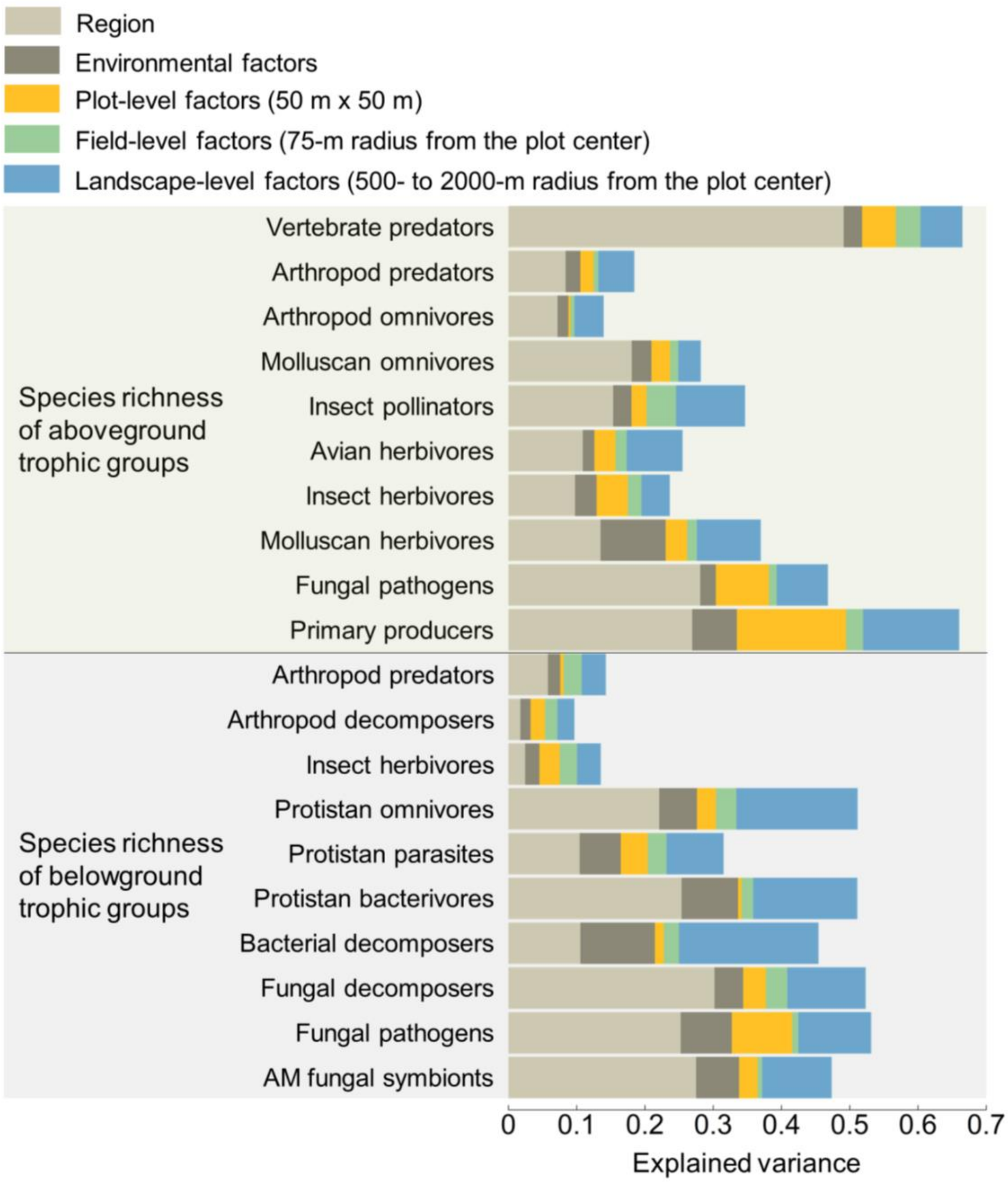

Supplementary figure 1. **Relative importance of land-use factors in explaining the species richness of multiple above- and belowground trophic groups *considering the region as a predictor in the models*.** Explained variance was calculated for each group of predictors: region in light brown, environmental factors in grey, plot-level (50 m × 50 m) factors in yellow, field-level (75-m radius from the plot center) factors in green, and landscape-level (500- to 2000-m

radius from the plot center) factors in blue. Note that the scale at which landscape land-use factors varies among trophic groups (see Fig. 4). All predictors and response variables were scaled to interpret parameter estimates on a comparable scale.

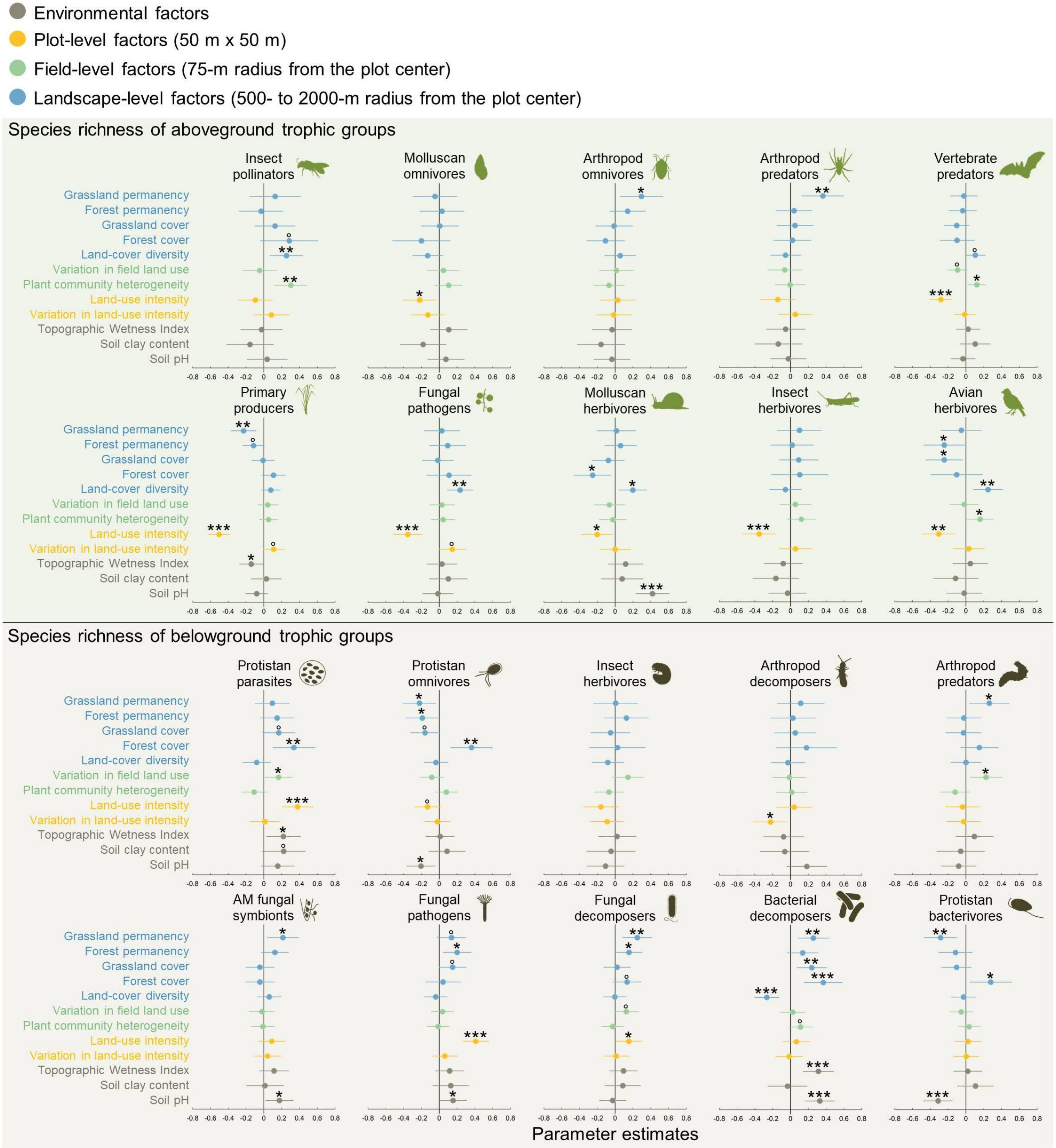

Supplementary figure 2. **Drivers of the species richness of multiple above- and belowground trophic groups *considering the region as a predictor in the models*.** The parameter estimates of

the predictor 'Region' is not shown as it is a categorical predictor and their coefficients are not straightforward to interpret. Data are presented as the parameter estimates (standardized regression coefficients) from linear models and we show the 95 % confidence intervals associated with the parameter estimates. Grey points show the parameter estimates of each environmental factor. Yellow points show the parameter estimates of plot-level factors, green points show the parameter estimates of field-level factors; and blue points show the parameter estimates of landscape-level land-use factors. Note that the scale at which landscape land-use factors varies among trophic groups (see Fig. 4). All predictors were scaled to interpret parameter estimates on a comparable scale. Plot-level and landscape-level predictors were log-transformed. *P* values of the best selected models for each model parameter are given as: °$P < 0.10$; *$P < 0.05$;**$P < 0.01$;***$P < 0.001$. $n = 150$ biologically independent samples for belowground AM fungal symbionts, fungal pathogens, fungal decomposers, protistan bacterivores, protistan parasites, protistan omnivores, insect herbivores, arthropod predators and aboveground primary producers, avian herbivores; $n = 149$ biologically independent samples for aboveground vertebrate predators; $n = 148$ biologically independent samples for belowground bacterial decomposers; $n = 144$ biologically independent samples for aboveground fungal pathogens; $n = 139$ biologically independent samples for belowground arthropod decomposers and aboveground insect herbivores, arthropod omnivores, arthropod predators; $n = 134$ biologically independent samples for aboveground molluscan herbivores, molluscan omnivores; $n = 113$ biologically independent samples for aboveground insect pollinators.

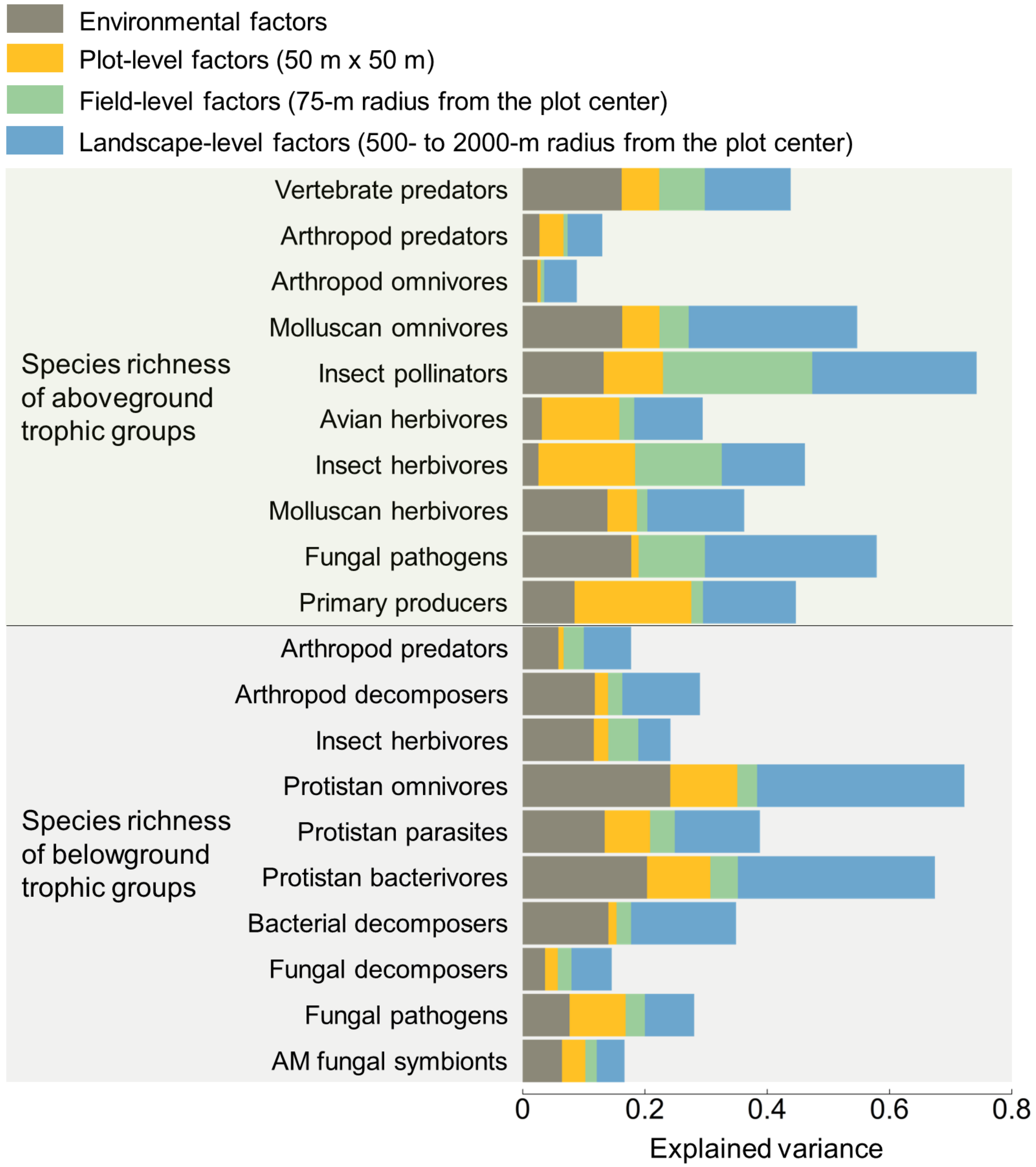

Supplementary figure 3. **Relative importance of land-use factors in explaining the species richness of multiple above- and belowground trophic groups, *considering a random subset of plots with non-overlapping buffers*.** The number of plots was n = 92, n = 65, n = 39 for the 500-m radius, 1000-m radius and 2000-m radius respectively. Relative effects of estimates were calculated for each group of predictors: environmental factors in grey, plot-level (50 m × 50 m)

factors in yellow, field-level (75-m radius from the plot center) factors in green, and landscape-level (500- to 2000-m radius from the plot center) factors in blue. All predictors and response variables were scaled to interpret parameter estimates on a comparable scale.

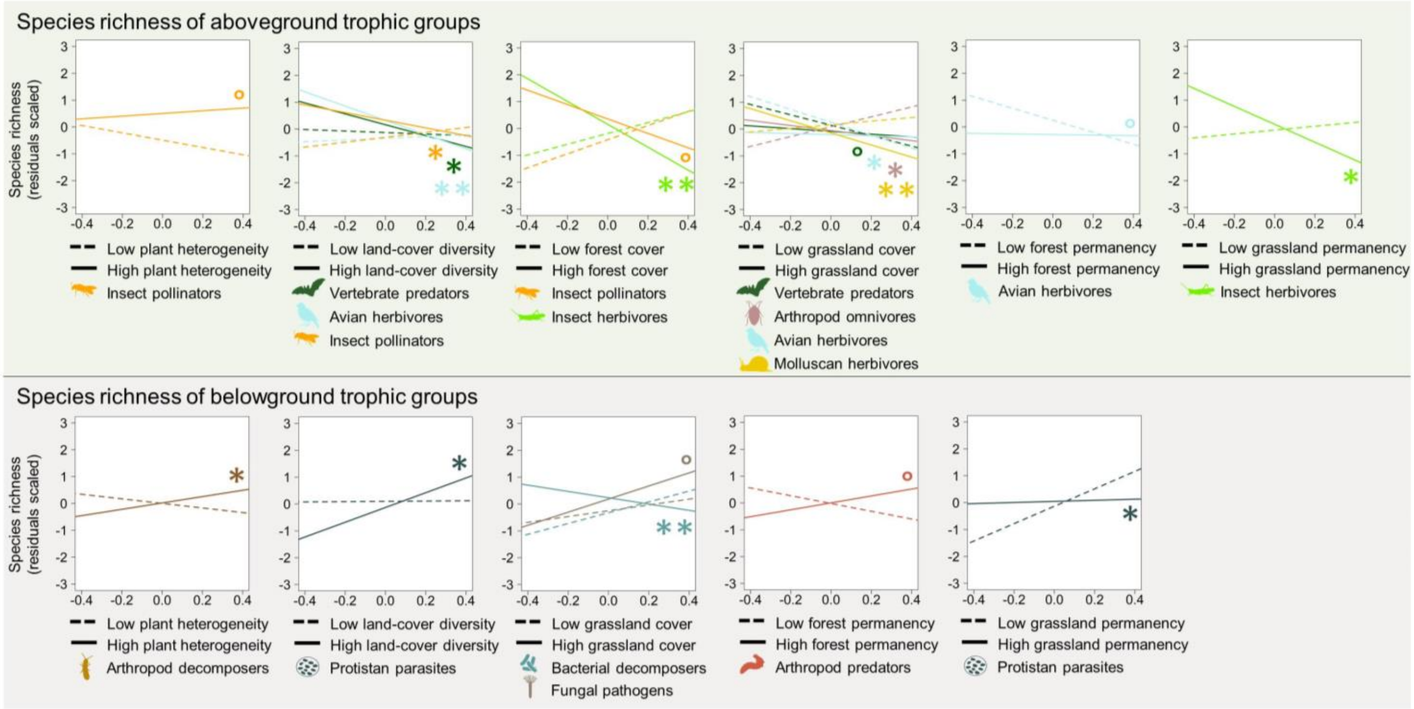

Supplementary figure 4. **Interactions between the plot land-use intensity and landscape land use.** Significant ($P < 0.05$) interactions are marked with an asterisk and marginally significant ($P < 0.10$) interactions are marked with a dot, with the colour of this denoting the respective trophic group. Aboveground species richness was higher in plots with low land-use intensity surrounded by heterogeneous plant communities ($P < 0.10$ for insect pollinators) and situated in landscapes with high land-cover diversity ($P < 0.05$ for insect pollinators and vertebrate predators, $P < 0.01$ for avian herbivores), high forest cover ($P < 0.10$ for insect pollinators and $P < 0.01$ for insect herbivores) and high grassland permanency ($P < 0.05$ for insect herbivores). However, plot land-use intensity had stronger negative effects on aboveground species richness in plots situated in these landscapes with diverse and permanent habitats. In habitat-rich landscapes, grassland species may particularly suffer from local land-use intensification, while generalist species present in simplified landscapes might cope with local land-use intensification[1,2]. By contrast, the species richness of belowground groups tend in general to be higher in plots with high land-use intensity surrounded by diverse habitats ($P < 0.05$ for the effect of field-plant heterogeneity for

arthropod decomposers, and $P < 0.05$ for the effect of land-cover diversity on protistan parasites), and in landscapes with high grassland ($P < 0.10$ for fungal pathogens) or forest cover ($P < 0.10$ for arthropod predators). Belowground communities might benefit from land-use intensity as it can increase soil resource availability, thus potentially enhancing the abundance of belowground groups, and the number of species detected[3–6]. This may be particularly true in landscapes with diverse habitats in the surroundings that can create spill-over[7,8].

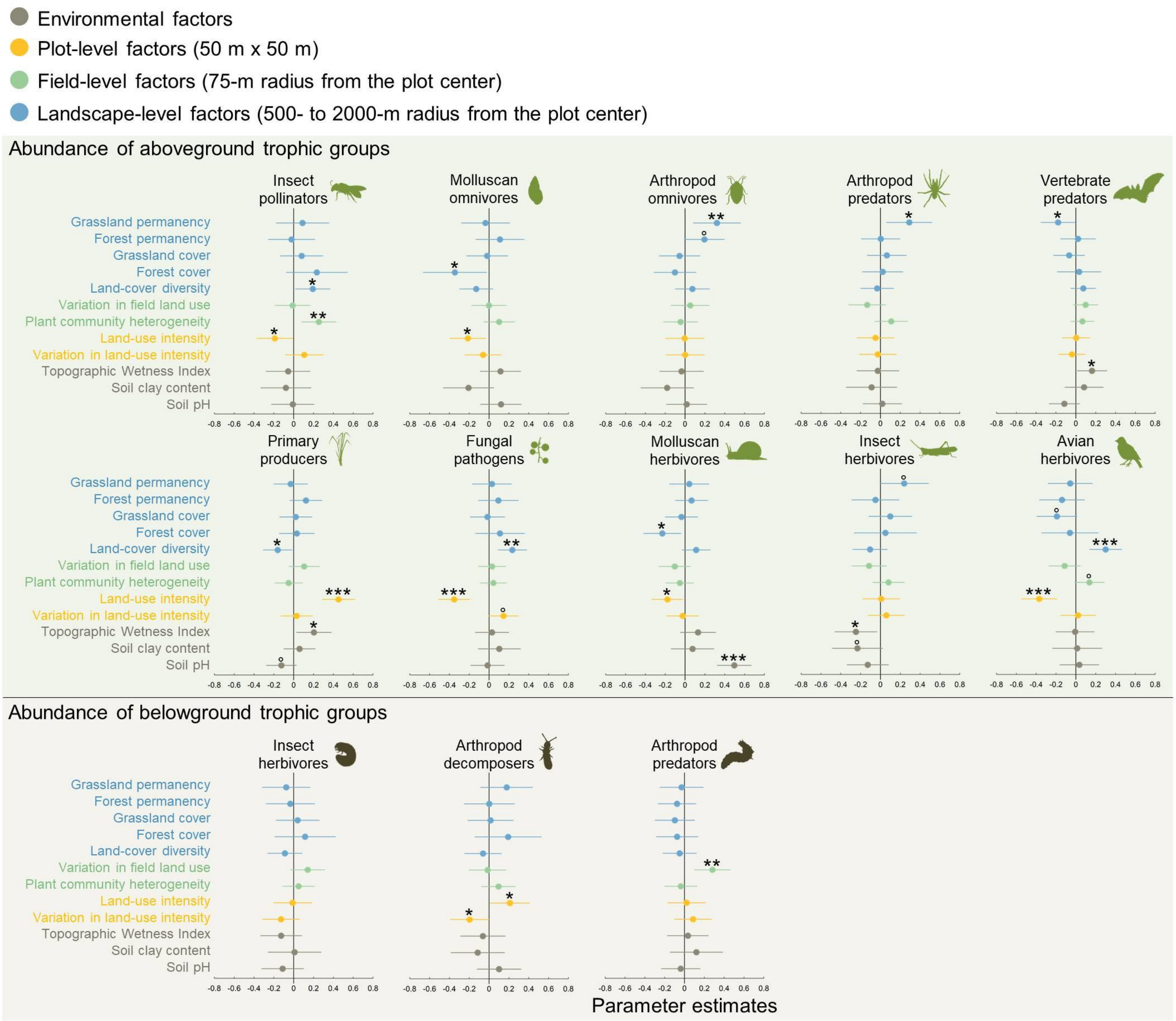

Supplementary figure 5. **Drivers of the *abundance* of multiple above- and belowground trophic groups.** For primary producers, we ran the analysis on the primary producer biomass (g.m^{-2}). We did not have data on the abundance of arbuscular mycorrhizal (AM) fungal symbiont, fungal pathogen, fungal decomposer, bacterial decomposer, protistan bacterivore, protistan omnivore and protistan parasite phylotypes in each plot, so they were excluded from this analysis. Data are presented as the parameter estimates (standardized regression coefficients)

from linear models and we show the 95 % confidence intervals associated with the parameter estimates. Grey points show the parameter estimates of each environmental factor. Yellow points show the parameter estimates of plot-level land-use factors, green points show the parameter estimates of field-level factors; and blue points show the parameter estimates of landscape-level land-use factors. Note that the scale at which landscape land-use factors varies among trophic groups (see Fig. 4). All predictors were scaled to interpret parameter estimates on a comparable scale. Plot-level and landscape-level land-use predictors were log-transformed. *P* values of the best selected models for each model parameter are given as: °$P < 0.10$; *$P < 0.05$;**$P < 0.01$;***$P < 0.001$. $n = 150$ biologically independent samples for belowground insect herbivores, arthropod predators and aboveground primary producers, avian herbivores; $n = 149$ biologically independent samples for aboveground vertebrate predators; $n = 144$ biologically independent samples for aboveground fungal pathogens; $n = 139$ biologically independent samples for belowground arthropod decomposers and aboveground insect herbivores, arthropod omnivores, arthropod predators; $n = 134$ biologically independent samples for aboveground molluscan herbivores, molluscan omnivores; $n = 113$ biologically independent samples for aboveground insect pollinators.

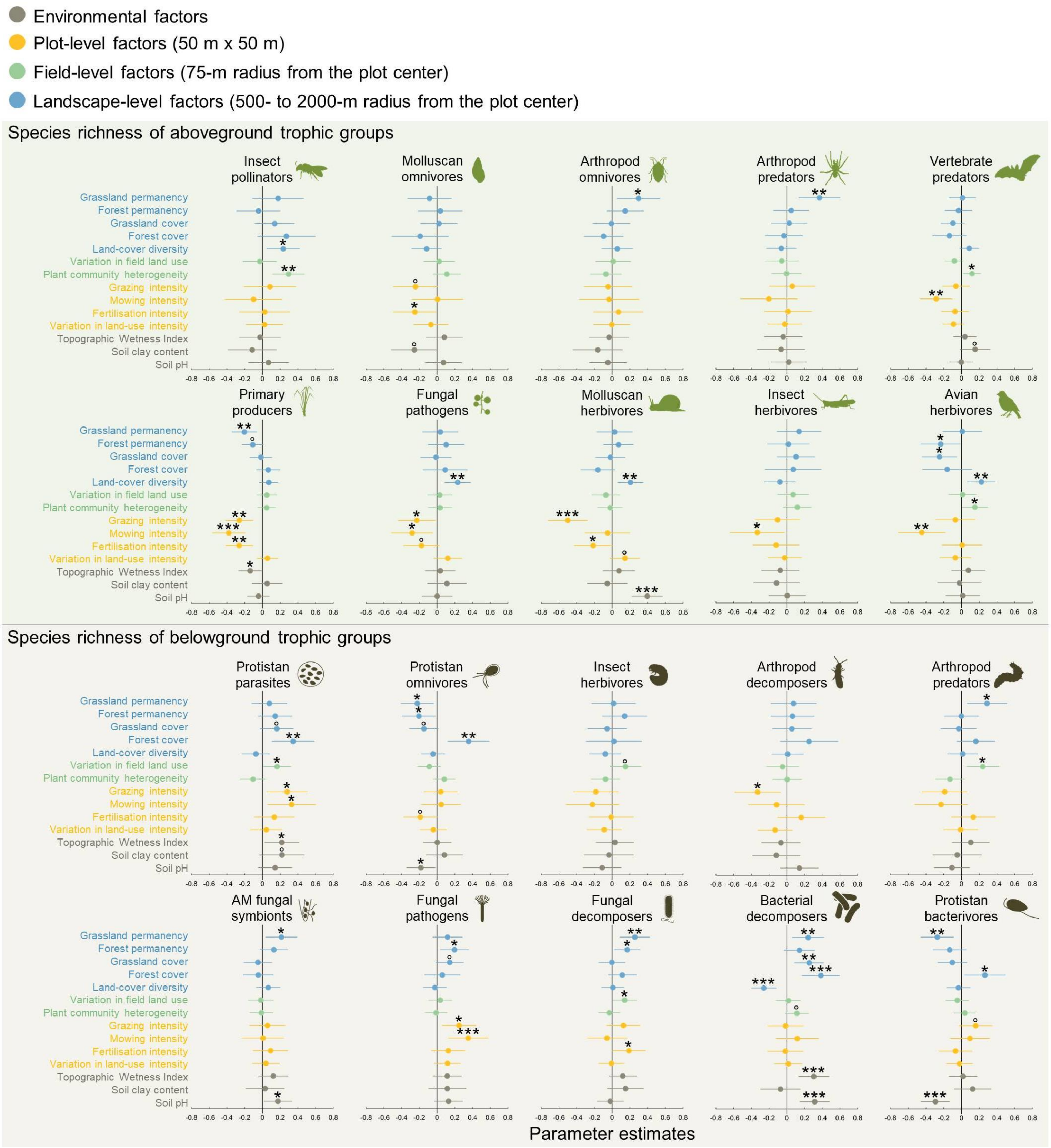

Supplementary figure 6. **Drivers of the species richness of multiple above- and belowground trophic groups** *considering each plot-level land-use component (i.e. grazing intensity, mowing*

intensity and fertilisation intensity) instead of the land-use intensity index. Data are presented as the parameter estimates (standardized regression coefficients) from linear models and we show the 95 % confidence intervals associated with the parameter estimates. Grey points show the parameter estimates of each environmental factor. Yellow points show the parameter estimates of plot-level land-use factors, green points show the parameter estimates of field-level factors; and blue points show the parameter estimates of landscape-level land-use factors. Note that the scale at which landscape land-use factors varies among trophic groups (see Fig. 4). All predictors were scaled to interpret parameter estimates on a comparable scale. Plot-level and landscape-level land-use predictors were log-transformed. *P* values of the best selected models for each model parameter are given as: °$P < 0.10$; *$P < 0.05$;**$P < 0.01$;***$P < 0.001$. $n = 150$ biologically independent samples for belowground AM fungal symbionts, fungal pathogens, fungal decomposers, protistan bacterivores, protistan parasites, protistan omnivores, insect herbivores, arthropod predators and aboveground primary producers, avian herbivores; $n = 149$ biologically independent samples for aboveground vertebrate predators; $n = 148$ biologically independent samples for belowground bacterial decomposers; $n = 144$ biologically independent samples for aboveground fungal pathogens; $n = 139$ biologically independent samples for belowground arthropod decomposers and aboveground insect herbivores, arthropod omnivores, arthropod predators; $n = 134$ biologically independent samples for aboveground molluscan herbivores, molluscan omnivores; $n = 113$ biologically independent samples for aboveground insect pollinators.

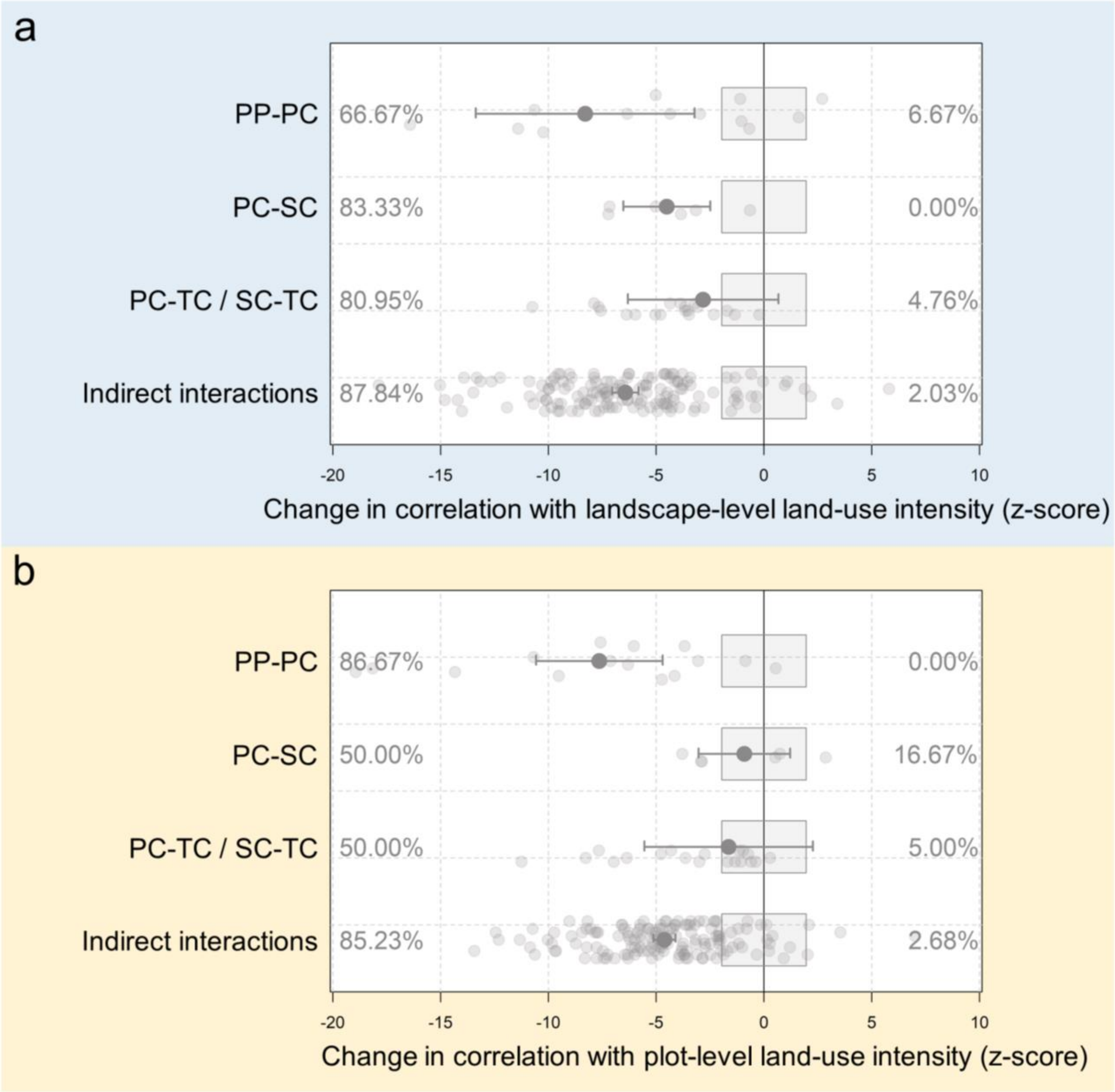

Supplementary figure 7. **Effect of the landscape and plot land-use intensity on correlations between the species richness of above- and belowground trophic levels.** Z-scores (standardized effect sizes) show the changes in Pearson-correlation strength (changes in r) between the species richness of pairs of trophic levels in (a) plots in low (n = 75 plots) and high (n = 75 plots) landscape land-use intensity or (b) plots in low (n = 75 plots) and high (n = 75 plots) plot land-use intensity. To calculate z-scores, we divided the 150 plots into 75 plots with the lowest landscape-level or plot-level land-use intensity and 75 plots with the highest

landscape-level or plot-level land-use intensity values, and calculated the differences in Pearson coefficient of correlation. We then compared these values to a distribution of simulated r-value differences (n = 999) in which we randomized the values of landscape or plot land-use intensity (low or high) between plots for each pair of trophic levels. On the basis of this random distribution PP, primary producers; PC, primary consumers; SC, secondary consumers; TC, tertiary consumers., z-scores and P values were calculated. All correlations were grouped into trophic levels: Positive z-scores indicate increases in correlation strength between the species richness of two trophic levels at high landscape or plot land-use intensity, and negative z-scores indicate decreases in correlation strengths between the species richness of two trophic levels at high landscape or plot land-use intensity. Each coloured dot represents one correlation; larger dots represent the mean and bars the 95 % confidence intervals. Coloured rectangles separate P value levels ($P < 0.05$ for dots outside the rectangle and not significant for dots inside). Percentages of positive and negative significant changes in correlation are indicated.

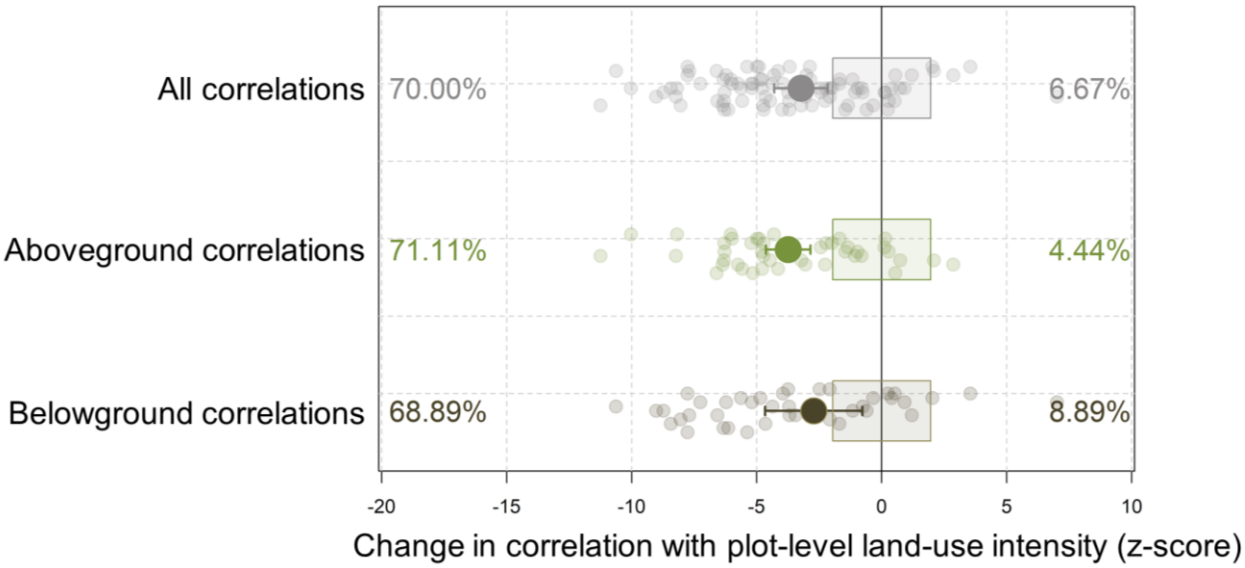

Supplementary figure 8. **Effect of the *plot land-use intensity* on correlations between the species richness of above- and belowground trophic groups.** Z-scores (standardized effect sizes) show the changes in Pearson-correlation strength (changes in r) between the species richness of pairs of trophic groups in plots in low (*n* = 75 plots) and high (*n* = 75 plots) plot land-use intensity. To calculate z-scores, we divided the 150 plots into 75 plots with the plot-level land-use intensity and 75 plots with the highest plot-level land-use intensity values, and calculated the differences in Pearson coefficient of correlation. We then compared these values to a distribution of simulated r-value differences (*n* = 999) in which we randomized the values of plot land-use intensity (low or high) between plots for each pair of trophic groups. On the basis of this random distribution, z-scores and *P* values were calculated. Positive z-scores indicate increases in correlation strength between the species richness of two trophic groups at high plot land-use intensity, and negative z-scores indicate decreases in correlation strengths between the species richness of two trophic groups at high plot land-use intensity. Each coloured dot represents one correlation; larger dots represent the mean and bars the 95 % confidence intervals. Coloured rectangles separate *P* value levels (*P* < 0.05 for dots outside the rectangle and not

significant for dots inside). Percentages of positive and negative significant changes in correlation are indicated.

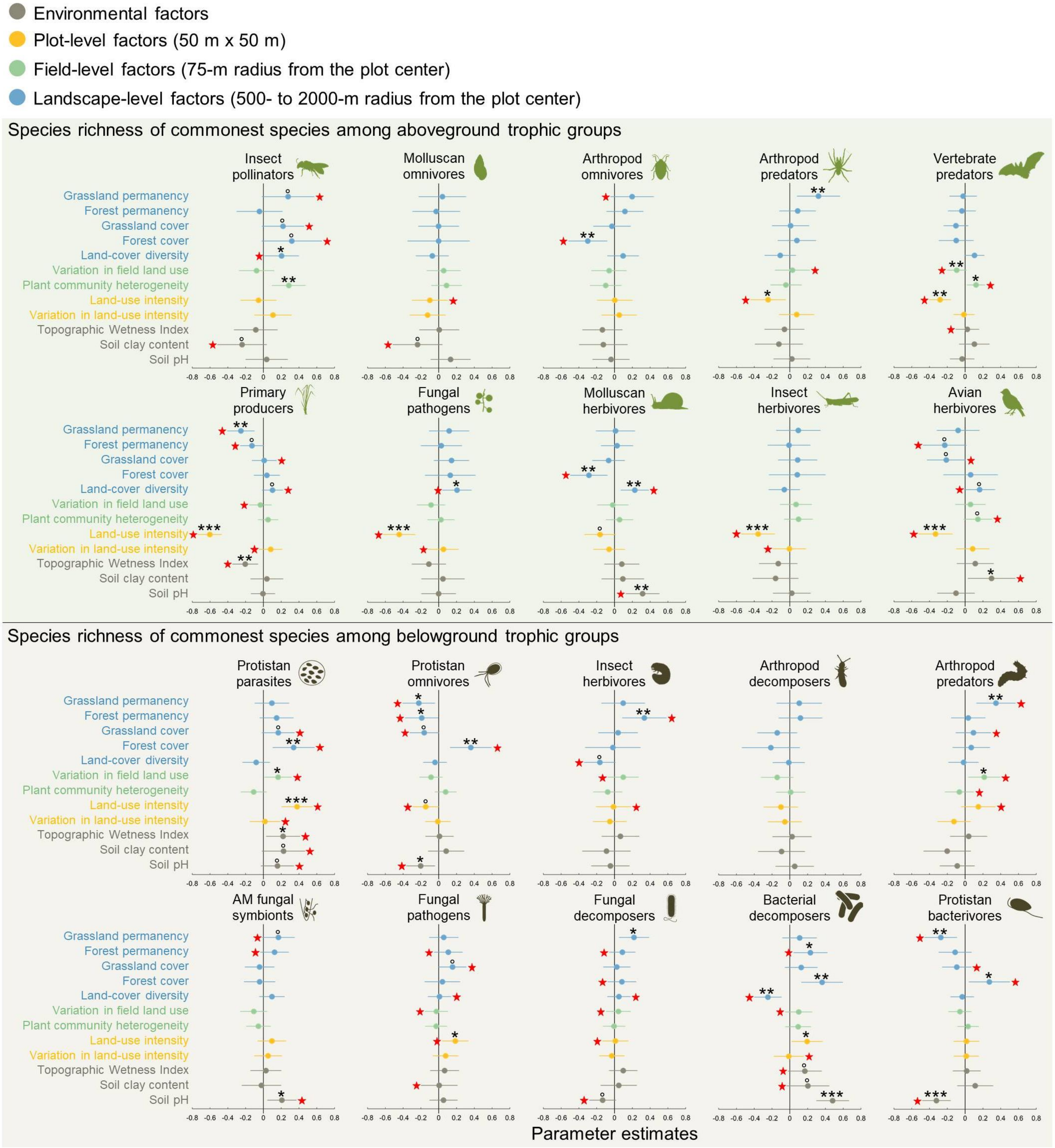

Supplementary figure 9. **Drivers of the species richness *among the commonest species* of multiple above- and belowground trophic groups.** Common species were the species

accounting for 80 % of the total occurrence. Red stars indicate differences in the significance of drivers between common and rare species (Supplementary Fig. 10). The red star was positioned on the left side if the predictor tended to have a more negative effect and on the right side if the predictor tended to have a more positive effect. Data are presented as the parameter estimates (standardized regression coefficients) from linear models and we show the 95 % confidence intervals associated with the parameter estimates. Grey points show the parameter estimates of each environmental factor. Yellow points show the parameter estimates of plot-level land-use factors, green points show the parameter estimates of field-level factors; and blue points show the parameter estimates of landscape-level land-use factors. Note that the scale at which landscape land-use factors varies among trophic groups (see Fig. 4). All predictors were scaled to interpret parameter estimates on a comparable scale. Plot-level and landscape-level land-use predictors were log-transformed. *P* values of the best selected models for each model parameter are given as: °$P < 0.10$; *$P < 0.05$;**$P < 0.01$;***$P < 0.001$. $n = 150$ biologically independent samples for belowground AM fungal symbionts, fungal pathogens, fungal decomposers, protistan bacterivores, protistan parasites, protistan omnivores, insect herbivores, arthropod predators and aboveground primary producers, avian herbivores; $n = 149$ biologically independent samples for aboveground vertebrate predators; $n = 148$ biologically independent samples for belowground bacterial decomposers; $n = 144$ biologically independent samples for aboveground fungal pathogens; $n = 139$ biologically independent samples for belowground arthropod decomposers and aboveground insect herbivores, arthropod omnivores, arthropod predators; $n = 134$ biologically independent samples for aboveground molluscan herbivores, molluscan omnivores; $n = 113$ biologically independent samples for aboveground insect pollinators.

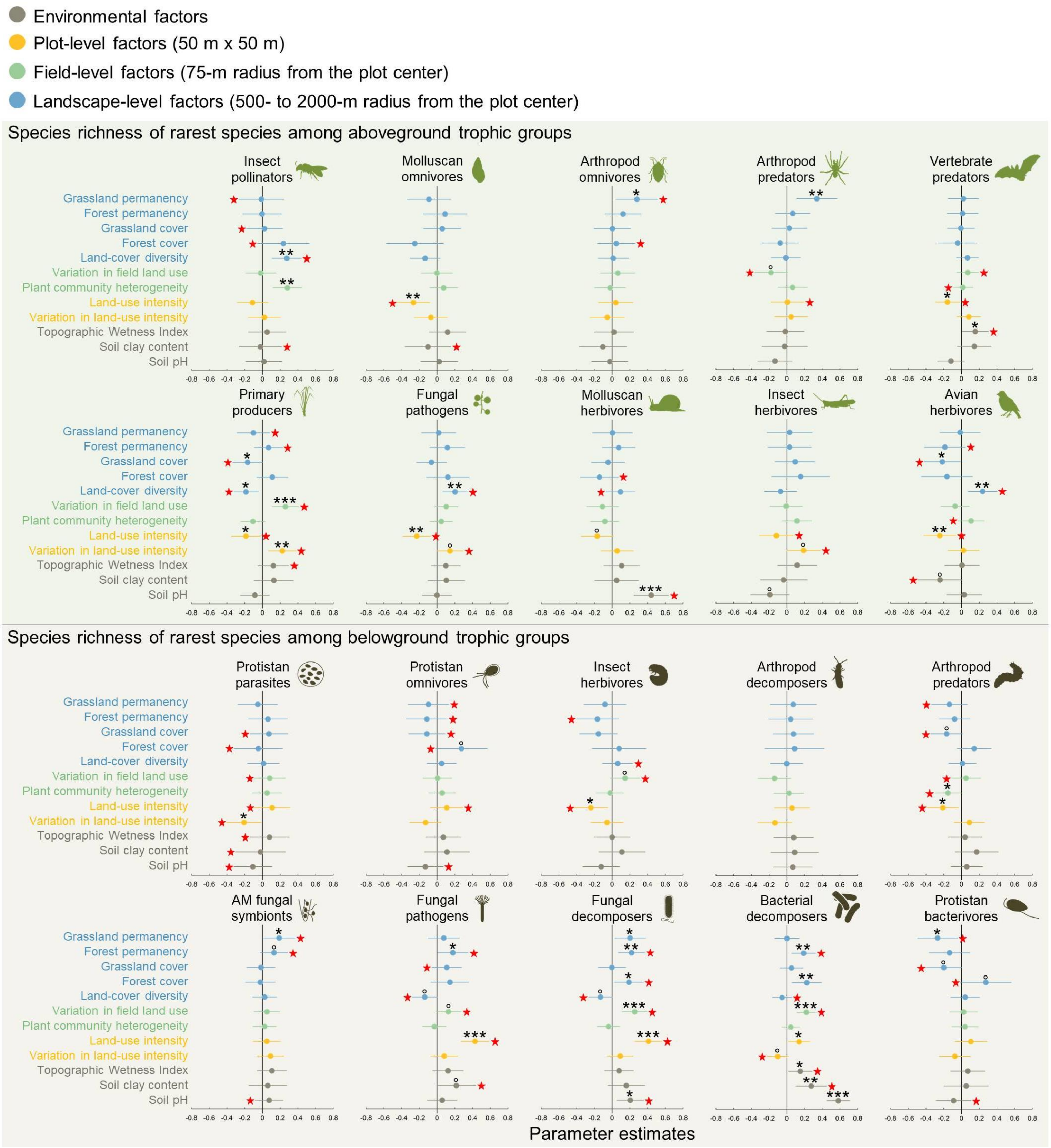

Supplementary figure 10. **Drivers of the species richness *among the rarest species* of multiple above- and belowground trophic groups.** Rare species were the species accounting for less

than 80 % of the total occurrence. Red stars indicate differences in significance between common (Supplementary Fig. 9) and rare species. The red star was positioned on the left side if the predictor tended to have a more negative effect and on the right side if the predictor tended to have a more positive effect. Data are presented as the parameter estimates (standardized regression coefficients) from linear models and we show the 95 % confidence intervals associated with the parameter estimates. Grey points show the parameter estimates of each environmental factor. Yellow points show the parameter estimates of plot-level land-use factors, green points show the parameter estimates of field-level factors; and blue points show the parameter estimates of landscape-level land-use factors. Note that the scale at which landscape land-use factors varies among trophic groups (see Fig. 4). All predictors were scaled to interpret parameter estimates on a comparable scale. Plot-level and landscape-level land-use predictors were log-transformed. *P* values of the best selected models for each model parameter are given as: °$P < 0.10$; *$P < 0.05$;**$P < 0.01$;***$P < 0.001$. $n = 150$ biologically independent samples for belowground AM fungal symbionts, fungal pathogens, fungal decomposers, protistan bacterivores, protistan parasites, protistan omnivores, insect herbivores, arthropod predators and aboveground primary producers, avian herbivores; $n = 149$ biologically independent samples for aboveground vertebrate predators; $n = 148$ biologically independent samples for belowground bacterial decomposers; $n = 144$ biologically independent samples for aboveground fungal pathogens; $n = 139$ biologically independent samples for belowground arthropod decomposers and aboveground insect herbivores, arthropod omnivores, arthropod predators; $n = 134$ biologically independent samples for aboveground molluscan herbivores, molluscan omnivores; $n = 113$ biologically independent samples for aboveground insect pollinators.

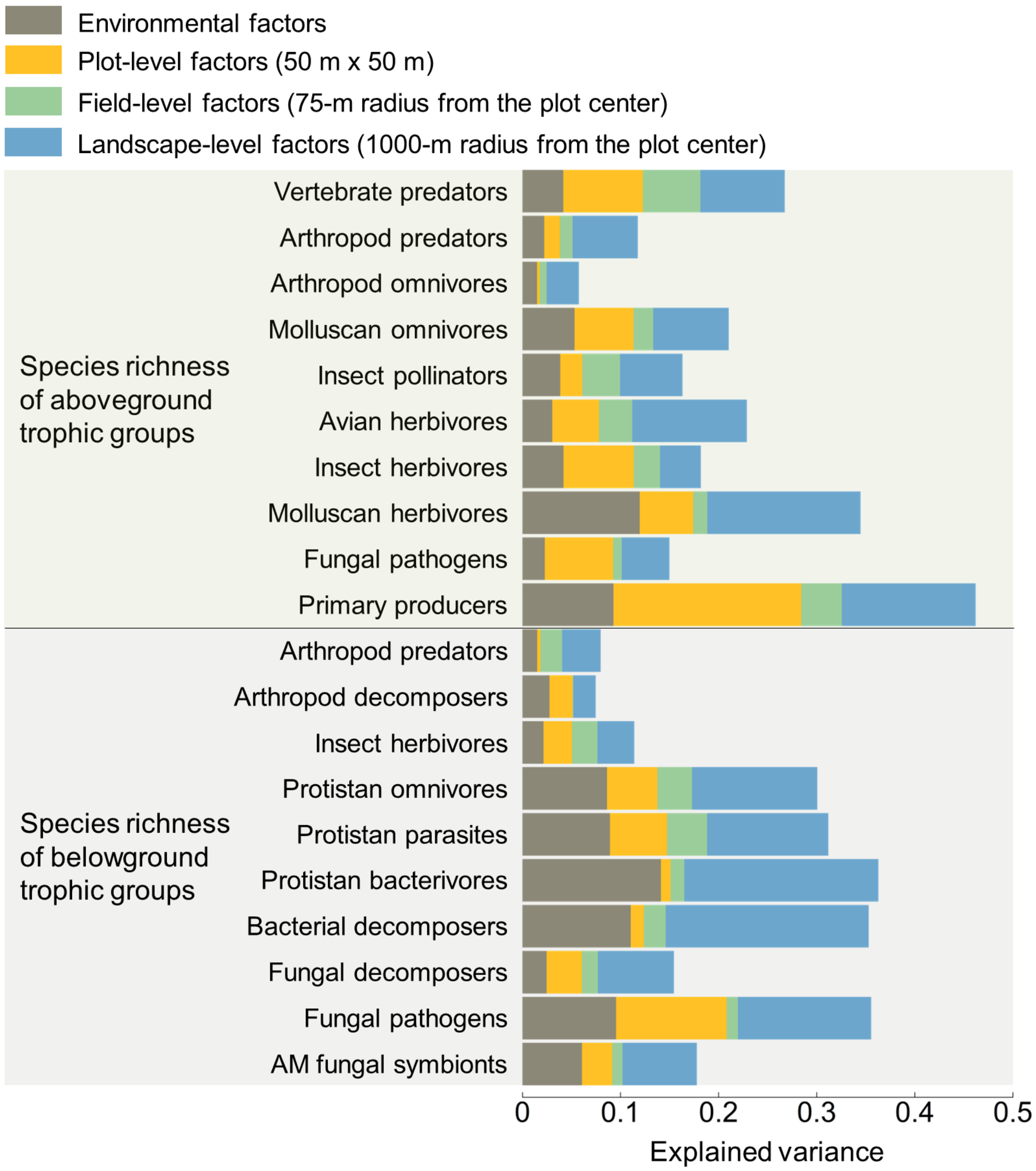

Supplementary figure 11. **Relative importance of land-use predictors in explaining the the species richness of multiple above- and belowground trophic groups, *considering a fixed 1000-m radius for all groups*.** Relative effects of estimates were calculated for each group of predictors: environmental covariates in grey, plot-level (50 m × 50 m) land-use predictors in yellow, field-level (75-m radius from the plot center) predictors in green, and landscape-level

(1000-m radius from the plot center) land-use predictors in blue. All predictors and response variables were scaled to interpret parameter estimates on a comparable scale.

Supplementary Table 1. **General mechanisms, adapted from metacommunity theory[9], driving the species richness of above- and belowground diversity, and their relation to the multiple predictors used in this study.** This table is not comprehensive but presents a selection of studies which support their use as predictors. Note also that these expectations are formulated for agroecosystems undergoing anthropogenic disturbances. Categories follow those of the general metacommunity theory of Thompson et al. 2020. For simplicity, and to retain consistency with Thompson et al. 2020, we separate abiotic and biotic drivers, although we acknowledge that abiotic conditions influence species interactions in nature.

Spatial scale considered	General mechanisms adapted from the metacommunity theory[9]		Predictors	Empirical evidence for effects on the species richness of aboveground trophic groups	Empirical evidence for effects on the species richness of belowground trophic groups
**Environmental factors**	Density-independent abiotic responses	Environmental conditions define the fundamental niche of species in which they are able to survive and reproduce, with certain conditions proving unsuitable for many species. Edaphic factors are major drivers of niche differentiation	Topographic Wetness Index	As TWI combines both upslope contributing area and slope then sites with a high TWI are likely to have wet soils that accumulate soil material via rainfall erosion and solifluction. TWI has been shown to be of use in predicting local plant species richness, depth to groundwater and soil pH and to be correlated to soil phosphorus and organic matter content[10–13]	The abundance and the diversity of belowground trophic groups can increase with TWI, since high TWI corresponds to wet soils that accumulate soil material[14,15]
			Soil clay content	Soil pH can affect the community composition among aboveground trophic groups such as insect herbivores, through its effects on primary producers or through direct effects[16–19]	Soil pH has been shown to be a primary determinant of the composition and diversity of soil communities[20]
			Soil pH	Plant species richness varies predictably with soil texture, which can in turn cascade up to the diversity of higher trophic levels[21,22]	The abundance and diversity of belowground trophic groups is strongly driven by soil clay content[4,23,24]
**Plot-level (local conditions)**	Density-independent abiotic responses	Perturbations caused by intensive land uses modify the fundamental niche of species, e.g. by generating physical disturbance, affecting abiotic conditions and resource availability.<br>In addition, temporal variation in land-use intensity can also create niches, allowing species with different strategies to coexist stably.	Land-use intensity	High levels of grassland land-use intensity decrease the diversity of multiple aboveground trophic groups, via direct effects of mowing, and changes to resource availability[25–28]	Land-use intensity affects the physical and chemical soil environment (e.g. soil compaction by livestock and machinery) which can reduce the abundance and diversity among belowground microbial or fungal communities. In addition, it affects resource availability for belowground groups[29–31]
			Variation in land-use intensity	High levels of interannual variation in land-use intensity increase the diversity of multiple aboveground groups[27]	Established belowground communities may be more sensitive to variation in land use, which can induce strong shifts in resource availability[28,31]
**Field-level (local conditions)**	Density-dependent biotic interactions	The abundance of competitors, mutualists, food species, pathogens and predators determine species survival. Plants are the basal organisms of the community, and their diversity shapes niche availability across trophic levels. This surrounding diversity enhances local diversity.	Plant community heterogeneity	Plant species richness is strongly correlated to the species richness of trophically-linked aboveground groups such as insect pollinators or herbivores. If interactions are specialized, then a higher diversity of plant species should support a higher diversity of another group. In addition, the composition of plant communities affects the habitat structure for multiple aboveground groups[22,25,32–34]. Plant species turnover is a strong determinant of species richness in agro-ecosystems[28,35,36]	The community composition of primary producers is a key driver of the community composition of multiple belowground trophic groups. It determines the quality and quantity of litter inputs to the soil[29,37–40]. Plant species turnover can affect the diversity among belowground trophic groups[28]

	Density-independent abiotic responses	Temporal variation in land use between years can create new niches, allowing species with different strategies to coexist stably.	Temporal variation in field land use	Past land use affects the current community composition of primary producers[41,42]	Land-use legacies are shaping the responses of belowground biodiversity. For example, the soil nutrient content reflects past land-use intensity and influences the effect of new nutrient inputs. In addition, plant communities are affected by past land use changes over time and these changes may in turn be manifested in belowground microbial communities that feed upon plant-derived soil organic matter[43,44]
**Landscape-level**	Dispersal processes	High dispersal maintains diversity in unsuitable habitat via population rescue and recolonization after extinction. High dispersal results from high local population density and successful movement (connectivity). The quantity and stability of semi-natural habitats, and the presence of a diversity of habitats in the surroundings enriches landscape diversity thus creating strong spill-over effects and enhances local diversity.	Grassland permanency	Increasing grassland cover permanency in a 1 km-landscape increases the local diversity of insect herbivores, insect pollinators and arthropod predators in agricultural grassland[1]	Habitat stability drives community assembly and affects the local species diversity of fungal decomposers[45]
			Forest permanency	In forests, the local diversity of primary producers and birds can increase with the forest age, indicating dispersal limitation[46–50]	In forests, local fungal diversity can increase with forest age[45,49]
			Grassland cover	Increasing grassland cover in the surrounding landscapes enhances local biodiversity of arthropods and birds[1,8,51,52]	Theoretical studies suggest that increasing the cover of semi-natural habitats such as grasslands in agricultural landscapes can increase local microbial diversity[53,54]
			Forest cover	In agricultural landscapes, species that occur in surrounding habitats such as forest can spill-over and enhance local biodiversity among arthropod and bird communities[8,55,56]	In forests, local fungal diversity increased with high surrounding forest cover and connectivity[57]
			Land-cover diversity	Increasing land-cover diversity increases the diversity of primary producers, insect pollinators, arthropod predators, and birds in agricultural landscapes; this effect can vary among taxa. For instance, it may be more positive for species that have lower habitat area requirements or higher habitat specialization levels[52,58]	The diversity of land-cover types in agricultural landscapes can affect the species pool of belowground microbes[53,54]

Supplementary Table 2. **Model selection based on second-order Akaike information criterion (AICc) for each trophic group.** Three competing models were fitted for each trophic group with the landscape land-use factors calculated either in a 500-m radius, 1000-m radius or 2000-m radius of the grassland plot. The model for which the second-order Akaike information criterion (AICc) was lowest was selected. When the AICc of the models were separated by a Δ AICc < 2, we retained the model with the largest spatial scale.

	Trophic group	AICc model considering 500-m radius landscape	AICc model considering 1000-m radius landscape	AICc model considering 2000-m radius landscape
**Species richness of aboveground trophic groups**	Vertebrate predators	293.661	291.387	289.985
	Arthropod predators	396.535	400.504	403.607
	Arthropod omnivores	403.966	407.450	408.203
	Molluscan omnivores	366.965	366.604	366.628
	Insect pollinators	306.170	307.531	299.357
	Avian herbivores	420.724	413.997	411.592
	Insect herbivores	387.003	386.993	387.380
	Molluscan herbivores	349.030	351.206	351.089
	Fungal pathogens	356.298	356.316	348.053
	Primary producers	293.810	302.541	301.761
**Species richness of belowground trophic groups**	Arthropod predators	432.658	434.689	437.423
	Arthropod decomposers	402.615	404.317	403.430
	Insect herbivores	434.105	434.990	433.958
	Protist omnivores	374.769	365.964	348.256
	Protist parasites	404.791	399.006	402.655
	Protist bacterivores	368.744	359.167	348.405
	Bacterial decomposers	358.946	360.511	371.335
	Fungal decomposers	344.673	351.734	358.327
	Fungal pathogens	341.369	342.085	346.227
	AM fungal symbionts	359.562	361.553	361.632

Supplementary Table 3. **Details of the sampling methods for each trophic group.** Species richness of all taxa was summed at the grassland plot level and over sampling occasions. Note that for some groups, the taxonomic unit was either families (belowground insect larvae), amplicon sequence variants (ASV: arbuscular mycorrhizal fungal symbionts, fungal pathogens, fungal decomposers and bacterial decomposers) or operational taxonomic units (OTU: protists).

	Trophic group	Number of plots	Sub-groups	Sampling method	Authors and references
Aboveground trophic groups	Vertebrate predators	149	Birds	Bird surveying data during breeding times (March-June), estimated by audio-visual point-counts, done in 2008-2012	Jung, Renner, Böhm, Tschapka[28,59,60]
		149	Bats	Acoustic recordings of bats with a Pettersson-D1000X bat detector, along two 24 min point-stop transect of 200 m, done in 2008-2010	Jung, Tschapka[28,60–64]
	Arthropod predators	139	Aranea, Coleoptera (partim), Hemiptera (partim), Neuroptera, Orthoptera (partim)	Sweep netting along transects of 150 m with 60 double sweeps, done twice per plot in 2008	Lange, Pašalić, Türke, Gossner, Weisser[28,60]
	Arthropod omnivores	139	Dermaptera, Dictyoptera, Hemiptera (partim), Opiliones	Sweep netting along transects of 150 m with 60 double sweeps, done twice per plot in 2008	Lange, Pašalić, Türke, Gossner, Weisser[28]
	Molluscan omnivores	134	Mollusca	Five surface samples per plot (20 cm × 20 cm, about 2 cm deep) were collected using a sharp knife, along with the herbaceous vegetation, mosses, litter and the upper soil layer in June 2017. Snail shells were collected by hand using a stereomicroscope. and subsequently determined to species level	Wehner, Blüthgen[65]
	Insect pollinators	113	Diptera, Hymenoptera, Coleoptera	Sweep netting along transects of 150 m with 60 double sweeps, done twice per plot in 2008. In addition, records of all flower visitors during 6 hours, in one 200 × 3 m transect per plot, done in May 2008	Klein, Weiner, Werner, Blüthgen[28,33,60]
			Lepidoptera	Sweep netting along transects of 300 m during 30 min, done three times per plot in 2008	Krauss, Börschig[66]
	Avian herbivores	150	Birds	Bird surveying data during breeding times (March-June), estimated by audio-visual point-counts, done in 2008-2012	Jung, Renner, Böhm, Tschapka[28,59,60]
	Insect herbivores	139	Hemiptera (partim), Coleoptera (partim) and Orthoptera (partim)	Sweep netting along transects of 150 m with 60 double sweeps, done twice per plot in 2008	Lange, Pašalić, Türke, Gossner, Weisser[28,60]
	Molluscan herbivores	134	Mollusca	Five surface samples per plot (20 cm × 20 cm, about 2 cm deep) were collected using a sharp knife, along with the herbaceous vegetation, mosses, litter and the upper soil layer in June 2017. Snail shells were collected by hand using a stereomicroscope. and subsequently determined to species level	Wehner, Blüthgen[65]
	Fungal pathogens	144	Plant pathogen fungi	Records of foliar fungal pathogens along four 25 m-transects, done in 2011	Blaser, Fischer[28,60]
	Primary producers	150	Vascular plants	Measurement of % cover in a 4×4 m subplot, done in 2008-2018	Boch, Heinze, Hölzel, Klaus, Kleinebecker, Müller, Prati, Socher, Fischer[28,60,67,68]

Supplementary Table 3. Cont.

	Trophic group	Number of plots	Sub-groups	Sampling method	Authors and references
Belowground trophic groups	Arthropod decomposers	139	Myriapoda	Kempson extraction from one soil core of 20 × 5 cm per plot, done in 2011	Birkhofer, Diekötter, Wolters[4]
			Coleoptera (partim)	Sweep netting along transects of 150 m with 60 double sweeps, done twice per plot in 2008-2010, and extracted from two soil cores of 20 x 10 cm per site, done in 2011	Lange, Paŝalić, Türke, Gossner, Weisser[28]
			Oribatida	Kempson extraction from four soil cores of 4.5 × 10 cm per plot, done in 2019	Baulechner, Wolters
			Collembola	Kempson extraction from four soil cores of 4.5 × 10 cm per plot, done in 2019	Baulechner, Wolters
	Arthropod predators	150	Myriapoda	Kempson extraction from one soil core of 20 ×5 cm per plot, done in 2011	Birkhofer, Diekötter, Wolters[4,28]
			Insect larvae of Asilidae, Bibionidae, Cantharidae, Carabidae, Ceratopogonidae, Dolichopodidae, Empididae, Hydrophilidae, Muscidae, Phryneidae, Psychodidae, Rhagionidae, Scatopsidae, Sciaridae, Staphylinidae, Tabanidae, Therevidae	Extracted from a heat/moisture gradient in one soil core of 20 x 5 cm per site, done in 2011 over 8 days	Sonnemann, Wurst[4,28,60]
	Insect herbivores	150	Insect larvae of Byrrhiidae, Cecidomyiidae, Chrysomelidae,Curculionidae, Elateridae, Hepialidae, Noctuidae, Pyralidae, Scarabaeidae, Stratiomyidae, Tipulidae	Extracted from a heat/moisture gradient in one soil core of 20 x 5 cm per site, done in 2011 over a period of eight days	Sonnemann, Wurst[28,60]
	Protistan omnivores	150	Cercozoa	Extracted from fourteen 10 x 5.3 cm soil cores of the A horizon homogenized after removal of root material, done in 2011 and 2017. 1 g of the bulk soil sample was used for DNA extraction and the analyses of the V4 region of the 18S rRNA gene amplified using eukaryotic specific primers. Soil DNA was extracted using the DNeasy PowerSoil Kit (Qiagen GmbH, Hilden, Germany). Sequences were filtered for (1) 100% forward primer match; (2) length ≥ 200–710 bp and (3) ambiguities (N). Traces were scanned for chimaeras, trimmed to 530 bp, dereplicated to group 100% identical amplicons, and singletons removed. Remaining sequences were treated as operational taxonomic units (OTUs) and aligned to the PR2 database using BLASTn (default parameters). One hit per sequence was retained. Only OTUs with 100% coverage and protist taxa (excluding Metazoa, Fungi and Streptophyta) were retained for analysis	Venter, Arndt, Bonkowski, Fiore-Donno[28,60]
	Protistan parasites	150	Endomyxa	Extracted from fourteen 10 x 5.3 cm soil cores of the A horizon homogenized after removal of root material, done in 2011 and 2017. 1 g of the bulk soil sample was used for DNA extraction and the analyses of the V4 region of the 18S rRNA gene amplified using eukaryotic specific primers. Soil DNA was extracted using the DNeasy PowerSoil Kit (Qiagen GmbH, Hilden, Germany). Sequences were filtered for (1) 100% forward primer match; (2) length ≥ 200–710 bp and (3) ambiguities (N). Traces were scanned for chimaeras, trimmed to 530 bp, dereplicated to group 100% identical amplicons, and singletons removed. Remaining sequences were treated as operational taxonomic units (OTUs) and aligned to the PR2 database using BLASTn (default parameters). One hit per sequence was retained. Only OTUs with 100% coverage and protist taxa (excluding Metazoa, Fungi and Streptophyta) were retained for analysis	Venter, Arndt, Bonkowski, Fiore-Donno[28,60]

Supplementary Table 3. Cont.

	Trophic group	Number of plots	Sub-groups	Sampling method	Authors and references
Belowground trophic groups	Protistan bacterivores	150	Cercozoa	Extracted from fourteen 10 x 5.3 cm soil cores of the A horizon homogenized after removal of root material, done in 2011 and 2017. 1 g of the bulk soil sample was used for DNA extraction and the analyses of the V4 region of the 18S rRNA gene amplified using eukaryotic specific primers. Soil DNA was extracted using the DNeasy PowerSoil Kit (Qiagen GmbH, Hilden, Germany). Sequences were filtered for (1) 100% forward primer match; (2) length ≥ 200–710 bp and (3) ambiguities (N). Traces were scanned for chimaeras, trimmed to 530 bp, dereplicated to group 100% identical amplicons, and singletons removed. Remaining sequences were treated as operational taxonomic units (OTUs) and aligned to the PR2 database using BLASTn (default parameters). One hit per sequence was retained. Only OTUs with 100% coverage and protist taxa (excluding Metazoa, Fungi and Streptophyta) were retained for analysis.	Venter, Arndt, Bonkowski, Fiore-Donno[28,60]
	Bacterial decomposers	148	Soil bacteria	Extracted from fourteen 10 x 5.3 cm soil cores of the A horizon homogenized after removal of root material, done in 2011. 10 g of the homogenized soil was put immediately on liquid nitrogen and stored until RNA extraction. RNA was extracted using a custom protocol (Lueders protocol). Total RNA was isolated from soils and reverse transcribed into cDNA. Amplicons of the V3 region of the 16S rRNA gene were sequenced on an Illumina Hiseq platform using universal bacterial primers.	Baumgartner, Sikorski, Goldmann, Overmann[60,69–72]
	Fungal decomposers	150	Ascomycota , Basidiomycota, Chytridiomycota, Entomophthoromycota, Kickxellomycota, Monoblepharomycota, Mortierellomycota, Mucoromycota	Extracted from fourteen 10 x 5.3 cm soil cores of the A horizon homogenized after removal of root material, done in 2011, 2014 and 2017. Total microbial DNA was isolated from the bulk soil sample using a MoBioPowerSoil DNA Isolation Kit. A PCR approach was used to amplify fungal ITS-rDNA by using the primer pair ITS4/fITS7, containing the Illumina adapter sequences. PCR products were then purified, cleaned and sequenced using Illumina MiSeq.	Klemmer, Wubet, Buscot, Goldmann[73,74]
	Fungal pathogens	150	Ascomycota, Basidiomycota, Chytridiomycota, Entorrhizomycota, Olpidiomycota	Extracted from fourteen 10 x 5.3 cm soil cores of the A horizon homogenized after removal of root material, done in 2011, 2014 and 2017. Total microbial DNA was isolated from the bulk soil sample using a MoBioPowerSoil DNA Isolation Kit. A PCR approach was used to amplify fungal ITS-rDNA by using the primer pair ITS4/fITS7, containing the Illumina adapter sequences. PCR products were then purified, cleaned and sequenced using Illumina MiSeq.	Klemmer, Wubet, Buscot, Goldmann[73,74]
	AM fungal symbionts	150	Ascomycota, Basidiomycota, Glomeromycota, Mucoromycota	Extracted from fourteen 10 x 5.3 cm soil cores of the A horizon homogenized after removal of root material, done in 2011, 2014 and 2017. Total microbial DNA was isolated from the bulk soil sample using a MoBioPowerSoil DNA Isolation Kit. A PCR approach was used to amplify fungal ITS-rDNA by using the primer pair ITS4/fITS7, containing the Illumina adapter sequences. PCR products were then purified, cleaned and sequenced using Illumina MiSeq.	Klemmer, Wubet, Buscot, Goldmann[73,74]

Supplementary Table 4. Current average proportion of the different land-cover types, and past average proportion of grasslands and forests within a 2-km landscape of each grassland plot in the three Biodiversity Exploratories region.

			**Schwäbische Alb**	**Hainich National Park**	**Schorfheide-Chorin**
**Current landscape land use**	% croplands		14.81	38.34	27.77
	% grasslands		32.60	23.67	36.66
	% forests		44.07	32.29	26.29
	% roads		0.51	0.62	0.71
	% urban areas		7.98	5.03	4.89
	% water bodies		0.03	0.05	3.68
**Past landscape land use**	% grasslands	year 1820/50	30.34	8.60	27.36
		year 1910/30	26.56	5.97	25.50
		year 1960	30.82	7.64	22.45
	% forests	year 1820/50	32.22	21.82	20.68
		year 1910/30	35.49	26.57	18.82
		year 1960	37.37	29.50	22.46

Supplementary Table 5. Results of the variance inflation factor (VIF) analysis. VIF was performed to evaluate the risk of multicollinearity in the best selected models for the analyses of species richness among multiple above- and belowground trophic groups. No VIF values were > 10, indicating that there is no multicollinearity in the models.

	Trophic group	R^2	VIF
**Species richness of aboveground trophic groups**	Vertebrate predators	0.274	1.378
	Arthropod predators	0.143	1.166
	Arthropod omnivores	0.081	1.088
	Molluscan omnivores	0.210	1.266
	Insect pollinators	0.223	1.286
	Avian herbivores	0.241	1.318
	Insect herbivores	0.180	1.219
	Molluscan herbivores	0.355	1.551
	Fungal pathogens	0.197	1.246
	Primary producers	0.492	1.970
**Species richness of belowground trophic groups**	Arthropod predators	0.092	1.101
	Arthropod decomposers	0.081	1.088
	Insect herbivores	0.120	1.136
	Protist omnivores	0.378	1.609
	Protist parasites	0.312	1.453
	Protist bacterivores	0.407	1.686
	Bacterial decomposers	0.353	1.545
	Fungal decomposers	0.193	1.239
	Fungal pathogens	0.355	1.551
	AM fungal symbionts	0.189	1.232

Supplementary references

1. Le Provost, G. *et al.* Land-use history impacts functional diversity across multiple trophic groups. *Proc. Natl. Acad. Sci.* **117**, 1573–1579 (2020).

2. Gámez-Virués, S. *et al.* Landscape simplification filters species traits and drives biotic homogenization. *Nat. Commun.* **6**, 1–8 (2015).

3. Shange, R. S., Ankumah, R. O., Ibekwe, A. M., Zabawa, R. & Dowd, S. E. Distinct soil bacterial communities revealed under a diversely managed agroecosystem. *PLOS ONE* **7**, e40338 (2012).

4. Birkhofer, K. *et al.* General relationships between abiotic soil properties and soil biota across spatial scales and different land-use types. *PLOS ONE* **7**, e43292 (2012).

5. Poulsen, P. H. B. *et al.* Effects of fertilization with urban and agricultural organic wastes in a field trial – Prokaryotic diversity investigated by pyrosequencing. *Soil Biol. Biochem.* **57**, 784–793 (2013).

6. Filazzola, A. *et al.* The effects of livestock grazing on biodiversity are multi-trophic: a meta-analysis. *Ecol. Lett.* **23**, 1298–1309 (2020).

7. Blitzer, E. J. *et al.* Spillover of functionally important organisms between managed and natural habitats. *Agric. Ecosyst. Environ.* **146**, 34–43 (2012).

8. Tscharntke, T. *et al.* Landscape moderation of biodiversity patterns and processes-eight hypotheses. *Biol. Rev.* **87**, 661–685 (2012).

9. Thompson, P. L. *et al.* A process-based metacommunity framework linking local and regional scale community ecology. *Ecol. Lett.* https://doi.org/10.1111/ele.13568

10. Gessler, P. E., Moore, I. D., McKENZIE, N. J. & Ryan, P. J. Soil-landscape modelling and spatial prediction of soil attributes. *Int. J. Geogr. Inf. Syst.* **9**, 421–432.

11. Zinko, U., Seibert, J., Dynesius, M. & Nilsson, C. Plant species numbers predicted by a topography-based groundwater flow index. *Ecosystems* **8**, 430–441 (2005).

12. Sørensen, R., Zinko, U. & Seibert, J. On the calculation of the topographic wetness index: evaluation of different methods based on field observations. *Hydrol. Earth Syst. Sci.* 13 (2006).

13. Moeslund, J. E. *et al.* Topographically controlled soil moisture drives plant diversity patterns within grasslands. *Biodivers. Conserv.* **22**, 2151–2166 (2013).

14. Fierer, N. & Jackson, R. B. The diversity and biogeography of soil bacterial communities. *Proc. Natl. Acad. Sci.* **103**, 626–631 (2006).

15. Ågren, A. M., Lidberg, W., Strömgren, M., Ogilvie, J. & Arp, P. A. Evaluating digital terrain indices for soil wetness mapping–a Swedish case study. *Hydrol. Earth Syst. Sci.* **18**, (2014).

16. Keddy, P. A. Assembly and response rules: two goals for predictive community ecology. *J. Veg. Sci.* **3**, 157–164 (1992).

17. Löbel, S., Dengler, J. & Hobohm, C. Species richness of vascular plants, bryophytes and lichens in dry grasslands: The effects of environment, landscape structure and competition. *Folia Geobot.* **41**, 377–393 (2006).

18. Myers, M. C., Mason, J. T., Hoksch, B. J., Cambardella, C. A. & Pfrimmer, J. D. Birds and butterflies respond to soil-induced habitat heterogeneity in experimental plantings of

tallgrass prairie species managed as agroenergy crops in Iowa, USA. *J. Appl. Ecol.* **52**, 1176–1187 (2015).

19. Carvalheiro, L. G. *et al.* Soil eutrophication shaped the composition of pollinator assemblages during the past century. *Ecography* **43**, 209–221 (2020).

20. Delgado-Baquerizo, M. & Eldridge, D. J. Cross-biome drivers of soil bacterial alpha diversity on a worldwide scale. *Ecosystems* **22**, 1220–1231 (2019).

21. Barnes, P. W. & Harrison, A. T. Species distribution and community organization in a Nebraska Sandhills mixed prairie as influenced by plant/soil-water relationships. *Oecologia* **52**, 192–201 (1982).

22. Kéfi, S. *et al.* More than a meal… integrating non-feeding interactions into food webs. *Ecol. Lett.* **15**, 291–300 (2012).

23. Xue, P.-P., Carrillo, Y., Pino, V., Minasny, B. & McBratney, A. B. Soil properties drive microbial community structure in a large scale transect in South Eastern Australia. *Sci. Rep.* **8**, 11725 (2018).

24. Bickel, S. & Or, D. Soil bacterial diversity mediated by microscale aqueous-phase processes across biomes. *Nat. Commun.* **11**, 116 (2020).

25. Manning, P. *et al.* Grassland management intensification weakens the associations among the diversities of multiple plant and animal taxa. *Ecology* **96**, 1492–1501 (2015).

26. Benton, T. G., Bryant, D. M., Cole, L. & Crick, H. Q. Linking agricultural practice to insect and bird populations: a historical study over three decades. *J. Appl. Ecol.* **39**, 673–687 (2002).

27. Allan, E. *et al.* Interannual variation in land-use intensity enhances grassland multidiversity. *Proc. Natl. Acad. Sci.* **111**, 308–313 (2014).

28. Gossner, M. M. *et al.* Land-use intensification causes multitrophic homogenization of grassland communities. *Nature* **540**, 266–269 (2016).

29. De Vries, F. T. *et al.* Abiotic drivers and plant traits explain landscape-scale patterns in soil microbial communities. *Ecol. Lett.* **15**, 1230–1239 (2012).

30. Bardgett, R. D. *et al.* Soil microbial community patterns related to the history and intensity of grazing in sub-montane ecosystems. *Soil Biol. Biochem.* **33**, 1653–1664 (2001).

31. Boeddinghaus, R. S. *et al.* Plant functional trait shifts explain concurrent changes in the structure and function of grassland soil microbial communities. *J. Ecol.* **107**, 2197–2210 (2019).

32. Scherber, C. *et al.* Bottom-up effects of plant diversity on multitrophic interactions in a biodiversity experiment. *Nature* **468**, 553–556 (2010).

33. Weiner, C. N., Werner, M., Linsenmair, K. E. & Blüthgen, N. Land-use impacts on plant–pollinator networks: interaction strength and specialization predict pollinator declines. *Ecology* **95**, 466–474 (2014).

34. Deraison, H., Badenhausser, I., Börger, L. & Gross, N. Herbivore effect traits and their impact on plant community biomass: an experimental test using grasshoppers. *Funct. Ecol.* **29**, 650–661 (2015).

35. Clough, Y. *et al.* Alpha and beta diversity of arthropods and plants in organically and conventionally managed wheat fields. *J. Appl. Ecol.* **44**, 804–812 (2007).

36. Kessler, M. *et al.* Alpha and beta diversity of plants and animals along a tropical land-use gradient. *Ecol. Appl.* **19**, 2142–2156 (2009).

37. Orwin, K. H. *et al.* Linkages of plant traits to soil properties and the functioning of temperate grassland. *J. Ecol.* **98**, 1074–1083 (2010).

38. Reich, P. B. The world-wide 'fast–slow' plant economics spectrum: a traits manifesto. *J. Ecol.* **102**, 275–301 (2014).

39. Wardle, D. A. *et al.* Ecological linkages between aboveground and belowground biota. *Science* **304**, 1629–1633 (2004).

40. Manning, P., Morrison, S. A., Bonkowski, M. & Bardgett, R. D. Nitrogen enrichment modifies plant community structure via changes to plant–soil feedback. *Oecologia* **157**, 661–673 (2008).

41. Dupouey, J. L., Dambrine, E., Laffite, J. D. & Moares, C. Irreversible impact of past land use on forest soils and biodiversity. *Ecology* **83**, 2978–2984 (2002).

42. Purschke, O. *et al.* Interactive effects of landscape history and current management on dispersal trait diversity in grassland plant communities. *J. Ecol.* **102**, 437–446 (2014).

43. Yao, H., He, Z. L., Wilson, Mj. & Campbell, C. D. Microbial biomass and community structure in a sequence of soils with increasing fertility and changing land use. *Microb. Ecol.* **40**, 223–237 (2000).

44. Perring, M. P. *et al.* Global environmental change effects on ecosystems: the importance of land-use legacies. *Glob. Change Biol.* **22**, 1361–1371 (2016).

45. Fukami, T. *et al.* Assembly history dictates ecosystem functioning: evidence from wood decomposer communities. *Ecol. Lett.* **13**, 675–684 (2010).

46. Moning, C. & Müller, J. Critical forest age thresholds for the diversity of lichens, molluscs and birds in beech (Fagus sylvatica L.) dominated forests. *Ecol. Indic.* **9**, 922–932 (2009).

47. Brunet, J., Fritz, Ö. & Richnau, G. Biodiversity in European beech forests-a review with recommendations for sustainable forest management. *Ecol. Bull.* 77–94 (2010).

48. Boch, S. *et al.* High plant species richness indicates management-related disturbances rather than the conservation status of forests. *Basic Appl. Ecol.* **14**, 496–505 (2013).

49. Spake, R., Ezard, T. H. G., Martin, P. A., Newton, A. C. & Doncaster, C. P. A meta-analysis of functional group responses to forest recovery outside of the tropics. *Conserv. Biol. J. Soc. Conserv. Biol.* **29**, 1695–1703 (2015).

50. Paillet, Y. *et al.* Snags and large trees drive higher tree microhabitat densities in strict forest reserves. *For. Ecol. Manag.* **389**, 176–186 (2017).

51. Clough, Y. *et al.* Density of insect-pollinated grassland plants decreases with increasing surrounding land-use intensity. *Ecol. Lett.* **17**, 1168–1177 (2014).

52. Sirami, C. *et al.* Increasing crop heterogeneity enhances multitrophic diversity across agricultural regions. *Proc. Natl. Acad. Sci.* **116**, 16442–16447 (2019).

53. Bell, T. & Tylianakis, J. M. Microbes in the Anthropocene: spillover of agriculturally selected bacteria and their impact on natural ecosystems. *Proc. R. Soc. B Biol. Sci.* **283**, 20160896 (2016).

54. Thakur, M. P. *et al.* Towards an integrative understanding of soil biodiversity. *Biol. Rev.* **95**, 350–364 (2020).

55. Magura, T., Tóthmérész, B. & Molnár, T. Forest edge and diversity: carabids along forest-grassland transects. *Biodivers. Conserv.* **10**, 287–300 (2001).

56. Le Provost, G. *et al.* Trait-matching and mass effect determine the functional response of herbivore communities to land-use intensification. *Funct. Ecol.* **31**, 1600–1611 (2017).

57. Vannette, R. L., Leopold, D. R. & Fukami, T. Forest area and connectivity influence root-associated fungal communities in a fragmented landscape. *Ecology* **97**, 2374–2383 (2016).

58. Burel, F., Butet, A., Delettre, Y. R. & De La Peña, N. M. Differential response of selected taxa to landscape context and agricultural intensification. *Landsc. Urban Plan.* **67**, 195–204 (2004).

59. Renner, S. C. *et al.* Temporal changes in randomness of bird communities across Central Europe. *PLoS ONE* **9**, (2014).

60. Soliveres, S. *et al.* Biodiversity at multiple trophic levels is needed for ecosystem multifunctionality. *Nature* **536**, 456–459 (2016).

61. Jung, K., Kaiser, S., Böhm, S., Nieschulze, J. & Kalko, E. K. V. Moving in three dimensions: effects of structural complexity on occurrence and activity of insectivorous bats in managed forest stands. *J. Appl. Ecol.* **49**, 523–531 (2012).

62. Denzinger, A., Siemers, B. M., Schaub, A. & Schnitzler, H.-U. Echolocation by the barbastelle bat, Barbastella barbastellus. *J. Comp. Physiol. A* **187**, 521–528 (2001).

63. Russo, D. & Jones, G. Identification of twenty-two bat species (Mammalia: Chiroptera) from Italy by analysis of time-expanded recordings of echolocation calls. *J. Zool.* **258**, 91–103 (2002).

64. Estrada-Villegas, S., Meyer, C. F. J. & Kalko, E. K. V. Effects of tropical forest fragmentation on aerial insectivorous bats in a land-bridge island system. *Biol. Conserv.* **143**, 597–608 (2010).

65. Wehner, K. *et al.* Land-use in Europe affects land snail assemblages directly and indirectly by modulating abiotic and biotic drivers. *Ecosphere* **10**, e02726 (2019).

66. Börschig, C., Klein, A.-M., von Wehrden, H. & Krauss, J. Traits of butterfly communities change from specialist to generalist characteristics with increasing land-use intensity. *Basic Appl. Ecol.* **14**, 547–554 (2013).

67. Socher, S. A. *et al.* Direct and productivity-mediated indirect effects of fertilization, mowing and grazing on grassland species richness. *J. Ecol.* **100**, 1391–1399 (2012).

68. Socher, S. A. *et al.* Interacting effects of fertilization, mowing and grazing on plant species diversity of 1500 grasslands in Germany differ between regions. *Basic Appl. Ecol.* **14**, 126–136 (2013).

69. Lueders, T., Manefield, M. & Friedrich, M. W. Enhanced sensitivity of DNA-and rRNA-based stable isotope probing by fractionation and quantitative analysis of isopycnic centrifugation gradients. *Environ. Microbiol.* **6**, 73–78 (2004).

70. Bartram, A. K., Lynch, M. D. J., Stearns, J. C., Moreno-Hagelsieb, G. & Neufeld, J. D. Generation of Multimillion-Sequence 16S rRNA Gene Libraries from Complex Microbial

Communities by Assembling Paired-End Illumina Reads. *Appl. Environ. Microbiol.* **77**, 3846–3852 (2011).

71. Vieira, S. *et al.* Bacterial colonization of minerals in grassland soils is selective and highly dynamic. *Environ. Microbiol.* **22**, 917–933 (2020).

72. Vieira, S. *et al.* Drivers of the composition of active rhizosphere bacterial communities in temperate grasslands. *ISME J.* **14**, 463–475 (2020).

73. Schloss, P. D. *et al.* Introducing mothur: Open-Source, Platform-Independent, Community-Supported Software for Describing and Comparing Microbial Communities. *Appl. Environ. Microbiol.* **75**, 7537–7541 (2009).

74. Morris, E. K. *et al.* Land use and host neighbor identity effects on arbuscular mycorrhizal fungal community composition in focal plant rhizosphere. *Biodivers. Conserv.* **22**, 2193–2205 (2013).
