# Supplementary material for: Contrasting responses of above- and belowground diversity to multiple components of land-use intensity
Source: Nat Commun. 2021 Jun 24;12:3918. doi: 10.1038/s41467-021-23931-1 (PMC8225671; doi:10.1038/s41467-021-23931-1)
Supplement: Supplementary file 2 — Description of Additional Supplementary Files [file 41467_2021_23931_MOESM2_ESM.docx]

File Name: Supplementary Data 1

Description: Best linear model results for the analyses of the species richness among multiple above- and belowground trophic groups

File Name: Supplementary Data 2

Description: Correlations among predictors included in the models (r). All predictors included in the models were not significantly correlated

File Name: Supplementary Data 3

Description: Results of the analysis testing the effect of increasing landscape land-use intensity on correlations between the species richness of above- and belowground trophic groups. Z-scores (standardized effect sizes) show the changes in Pearson-correlation strength (changes in r) between the species richness of pairs of trophic groups in plots in low (*n* = 75 plots) and high (*n* = 75 plots) landscape land-use intensity. To calculate z-scores, we divided the 150 plots into 75 plots with the lowest landscape-level land-use intensity and 75 plots with the highest landscape-level land-use intensity values, and calculated the differences in Pearson coefficient of correlation. We then compared these values to a distribution of simulated r-value differences (*n* = 999) in which we randomized the values of landscape land-use intensity (low or high) between plots for each pair of trophic groups. On the basis of this random distribution, z-scores and *P* values were calculated. Mean indicates the mean of the randomization per pair of trophic groups, sd indicates the standard deviation of the randomization per pair of trophic groups, r differences indicate the changes in r. AG = Aboveground, BG = Belowground, PP = primary producers, PC = primary consumers, SC = secondary consumers, TC = tertiary consumers
