# Supplementary material for: Contrasting responses of above- and belowground diversity to multiple components of land-use intensity
Source: Nat Commun. 2021 Jun 24;12:3918. doi: 10.1038/s41467-021-23931-1 (PMC8225671; doi:10.1038/s41467-021-23931-1)
Supplement: Supplementary file 6 — Reporting Summary [file 41467_2021_23931_MOESM6_ESM.pdf]

nature research

Corresponding author(s): Gaëtane Le Provost
Last updated by author(s): Apr 29, 2021

Reporting Summary

Nature Research wishes to improve the reproducibility of the work that we publish. This form provides structure for consistency and transparency in reporting. For further information on Nature Research policies, see our Editorial Policies and the Editorial Policy Checklist.

Statistics

For all statistical analyses, confirm that the following items are present in the figure legend, table legend, main text, or Methods section.

n/a	Confirmed	
☐	☒	The exact sample size (n) for each experimental group/condition, given as a discrete number and unit of measurement
☐	☒	A statement on whether measurements were taken from distinct samples or whether the same sample was measured repeatedly
☐	☒	The statistical test(s) used AND whether they are one- or two-sided<br>*Only common tests should be described solely by name; describe more complex techniques in the Methods section.*
☐	☒	A description of all covariates tested
☐	☒	A description of any assumptions or corrections, such as tests of normality and adjustment for multiple comparisons
☐	☒	A full description of the statistical parameters including central tendency (e.g. means) or other basic estimates (e.g. regression coefficient) AND variation (e.g. standard deviation) or associated estimates of uncertainty (e.g. confidence intervals)
☐	☒	For null hypothesis testing, the test statistic (e.g. F, t, r) with confidence intervals, effect sizes, degrees of freedom and P value noted<br>*Give P values as exact values whenever suitable.*
☒	☐	For Bayesian analysis, information on the choice of priors and Markov chain Monte Carlo settings
☐	☒	For hierarchical and complex designs, identification of the appropriate level for tests and full reporting of outcomes
☐	☒	Estimates of effect sizes (e.g. Cohen's d, Pearson's r), indicating how they were calculated

Our web collection on statistics for biologists contains articles on many of the points above.

Software and code

Policy information about availability of computer code

Data collection

The diversity of bats was quantified with acoustic recordings taken in real time with a Pettersson-D1000x bat detector (Pettersson Electronic AG, Uppsala, Sweden). Bat species identification was then conducted using the software Avisoft SAS Lab Pro, Version 5.0.24 and onward (Raimund Specht, Avisoft Bioacoustics, Berlin Germany).

To quantify the diversity of belowground arbuscular mycorrhizal fungal symbionts, fungal pathogens and fungal decomposers, soil samples were collected and DNA was extracted using 'MO BIO Power Soil DNA isolation kit' (MO BIO Laboratories, Carlsbad, CA, USA) following the manufacturer’s protocol. We then use a Illumina Hiseq platform for the sequencing, and sequence reads were processed using plugins available in the QIIME 2™ plattform (https://qiime2.org/, Version 2017.12). To quantify the diversity of bacterial decomposers, RNA was extracted using a custom protocol (Lueders protocol) (https://doi.org/10.1128/AEM.00019-16). To quantify the diversity of protists, DNA was extracted using the DNeasy PowerSoil Kit (Qiagen GmbH, Hilden, Germany) following the manufacturer's protocol.

We used a motor driven soil column cylinder with a diameter of 8.3 cm for the soil sampling (Eijkelkamp, Giesbeek, The Netherlands) to determine soil depth and soil pH.

Data analysis

Data analyses were conducted using R version 4.0.3 and QGIS v 3.6

For manuscripts utilizing custom algorithms or software that are central to the research but not yet described in published literature, software must be made available to editors and reviewers. We strongly encourage code deposition in a community repository (e.g. GitHub). See the Nature Research guidelines for submitting code & software for further information.

Data

Policy information about availability of data

All manuscripts must include a data availability statement. This statement should provide the following information, where applicable:

- Accession codes, unique identifiers, or web links for publicly available datasets
- A list of figures that have associated raw data
- A description of any restrictions on data availability

This work is based on data from several projects of the Biodiversity Exploratories programme (DFG Priority Program 1374). The data used for analyses are publicly available from the Biodiversity Exploratories Information System (https://doi.org/10.17616/R32P9Q), or will become publicly available after an embargo period of five years from the end of data assembly to give the owners and collectors of the data time to perform their analysis. Any other relevant data are available from the corresponding author upon reasonable request.

Field-specific reporting

Please select the one below that is the best fit for your research. If you are not sure, read the appropriate sections before making your selection.

☐ Life sciences ☐ Behavioural & social sciences ☒ Ecological, evolutionary & environmental sciences

For a reference copy of the document with all sections, see nature.com/documents/nr-reporting-summary-flat.pdf

Ecological, evolutionary & environmental sciences study design

All studies must disclose on these points even when the disclosure is negative.

Study description

The study was conducted as part of the long-term Biodiversity Exploratories project (www.biodiversity-exploratories.de) in three German regions: (i) the UNESCO Biosphere Reserve Schwäbische Alb in the low mountains of south-western Germany; (ii) the Hainich National Park and surrounding areas in hilly central Germany; and (iii) the UNESCO Biosphere Reserve Schorfheide-Chorin in the post-glacial lowlands of north-eastern Germany. The three regions differ in climate, geology and topography, but each is characterized by a gradient of grassland land-use intensity that is typical for large parts of temperate Europe. In each region, fifty plots (50 m × 50 m) were chosen in mesic grasslands by stratified random sampling from a total of 500 candidate plots on which initial vegetation, soil and land-use surveys were conducted. This ensured that the plots covered the whole range of land-use intensities and management types, while minimizing confounding factors such as spatial position or soil type (https://doi.org/10.1016/j.baae.2010.07.009). We assessed the influence of multiple elements of local- and landscape-level land use on the diversity of more than 4,000 above- and belowground taxa, spanning 20 trophic groups. At each plot, we measured the species or family richness of these trophic groups using standard methodology from 150 biologically independent samples, between 2008 and 2018. The 150 agricultural grassland plots vary strongly in their local land-use intensity (quantified as a compound indices based on grazing, mowing and fertilization intensity), and are situated in landscapes of varying complexity and management history. In summary, there are 150 replicates, nested within three regions, each containing 50 replicates.

Research sample

The sample unit is the community of a grassland plot, considering 20 trophic groups; ten aboveground trophic groups: primary producers, fungal pathogens, molluscan herbivores, insect herbivores, avian herbivores, insect pollinators, molluscan omnivores, arthropod omnivores, arthropod predators and vertebrate predators; and ten belowground trophic groups: arbuscular mycorrhizal (AM) fungal symbionts, fungal pathogens, fungal decomposers, bacterial decomposers, protistan bacterivores, protistan parasites, protistan omnivores, insect herbivores, arthropod decomposers and arthropod predators. It is characterized by measures of cover, species, family number, amplicon sequence variants or operational taxonomic units numbers, and abundance. A sample unit is considered to represent the population of the different trophic groups at our study sites which measured 50 m x 50 m. Data from different years and traps were pooled per plot.

Sampling strategy

We sampled vascular plants in an area of 4 m × 4 m on the 150 grassland plots, and estimated the percentage cover of each occurring species. Fungal pathogens were sampled in four transects of 25 m × 1 m per plot on the 150 grassland plots. We inspected all occurring vascular plant species for infested individuals, sampled them and later identified the pathogenic fungi to the species level. For sampling aboveground insect herbivores, pollinators, arthropod omnivores and predators we used sweep netting by conducting 60 double sweeps along three 50-m plot-border transects in 138 of the 150 grassland plots. Additionally, some insect pollinators were hand-collected during their visits on flowers, identified and individuals counted (Diptera and Hymenoptera), or recorded within 30 min along a 300-m transect (Lepidoptera) in 113 of the 150 grassland plots. Aboveground molluscan herbivores and omnivores were sampled in 134 of the 150 grassland plots by collecting five surface samples per plot (20 cm × 20 cm, about 2 cm deep), using a sharp knife, along with the herbaceous vegetation, mosses, litter and the upper soil layer. Avian herbivores and vertebrate predators were sampled by audio-visual point counts, at the centre of the respective grassland plot (50 m × 50 m) for birds and along two 200-m plot-border transects for bats in the 150 grassland plots.

To sample belowground AM fungal symbionts, fungal pathogens, fungal decomposers, bacterial decomposers and protists, fourteen soil cores (diameter 4.8 cm) were taken from a 20 m × 20 m subarea of each grassland plot, and soil from the upper 10 cm of the upper horizon was homogenized after removal of root material. The bulk sample was split into subsamples for the analyses of AM fungal symbionts (n = 150), fungal pathogens (n = 150), fungal decomposers (n = 150), bacterial decomposers (n = 148) and protistan bacterivores (n = 150), parasites (n = 150) and omnivores (n = 150). Belowground insect herbivores (n = 150), arthropod predators (n = 150), and arthropod decomposers (n = 139) were sampled by collecting two soil cores (diameter 20 cm, depth 10 cm) in the grassland plots. Belowground insect herbivores, arthropod predators and arthropod decomposers were extracted from the first core using a modified heat extraction system over a period of eight days. The second soil core was hand-sorted for soil macrofauna. In 2019, belowground arthropod decomposers were extracted as a composite sample from nine soil cores (diameter 4.5 cm, depth 10

cm) in 139 of the 150 grassland plots. A subsample of this composite sample was used to identify the major taxonomic groups to species level. In addition to soil extraction, some belowground arthropod decomposer species were sampled when sweep netting (60 double sweeps along three 150-m plot-border transects in 139 of the 150 grassland plots).

No statistical methods were used to predetermine sample size.

Data collection

The percentage cover of each vascular plant species was visually estimated and recorded on sheets of paper. Aboveground fungal pathogens were sampled in four transects of 25 m × 1 m per plot. We visually inspected all occurring vascular plant species for infested individuals, sampled them and later identified the pathogenic fungi to the species level. Aboveground molluscan herbivores and omnivores were sampled by collecting five surface samples per plot (20 cm × 20 cm, about 2 cm deep), using a sharp knife, along with the herbaceous vegetation, mosses, litter and the upper soil layer. For sampling aboveground insect herbivores, pollinators, arthropod omnivores and predators we used sweep netting by conducting 60 double sweeps along three 50-m plot-border transects. Additionally, some insect pollinators were hand-collected during their visits on flowers, identified and individuals counted (Diptera and Hymenoptera), or recorded within 30 min along a 300-m transect (Lepidoptera). Avian herbivores and vertebrate predators were sampled by audio-visual point counts, at the center of the respective grassland plot (50 m × 50 m) for birds and along two 200-m plot-border transects for bats. Acoustic recordings of bats were taken in real time with a Pettersson-D1000x bat detector (Pettersson Electronic AG, Uppsala, Sweden). Bat species identification was then conducted using the software Avisoft SAS Lab Pro, Version 5.0.24 and onward (Raimund Specht, Avisoft Bioacoustics, Berlin Germany).

To sample belowground AM fungal symbionts, fungal pathogens, fungal decomposers, bacterial decomposers and protists, fourteen soil cores (diameter 4.8 cm) were taken using a soil-corer from a 20 m × 20 m subarea of each grassland plot, and soil from the upper 10 cm of the upper horizon was homogenized after removal of root material. For AM fungal symbionts, fungal pathogens, fungal decomposers, and protists, DNA or RNA was extracted using 'MO BIO Power Soil DNA isolation kit' (MO BIO Laboratories, Carlsbad, CA, USA) following the manufacturer's protocol. We then use a Illumina Hiseq platform for the sequencing, and sequence reads were processed using plugins available in the QIIME 2™ plattform (https://qiime2.org/, Version 2017.12). For bacterial decomposers, RNA was extracted using a custom protocol (Lueders protocol) (https://doi.org/10.1128/AEM.00019-16). Soil samples for DNA extraction were transported in cooled boxes (approx. 4°C - 8°C) to the field lab where within few hours after soil excavation the soil was sieved and roots were removed. Soils were then frozen at -20°C. Soil samples for RNA extraction were flash-frozen directly after excavation either in liquid nitrogen or dry ice. Soil samples were shipped from the field labs to the institute laboratories on dry ice. Until nucleic acid extraction, soil samples were stored either at -20°C (for DNA extraction) or liquid nitrogen (for RNA extraction). The DNA and RNA nucleic acid extracts were stored at -20°C or -80°C, respectively. Belowground insect herbivores, arthropod predators, and arthropod decomposers were sampled by collecting two soil cores (diameter 20 cm, depth 10 cm) in the grassland plots. Belowground insect herbivores, arthropod predators and arthropod decomposers were extracted from the first core using a modified heat extraction system over a period of eight days. The second soil core was hand-sorted for soil macrofauna. In 2019, belowground arthropod decomposers were extracted as a composite sample from nine soil cores (diameter 4.5 cm, depth 10 cm). A subsample of this composite sample was used to identify the major taxonomic groups to species level. In addition to soil extraction, some belowground arthropod decomposer species were sampled when sweep netting (60 double sweeps along three 150-m plot-border transects).

Soil depth was measured as the combined thickness of all topsoil and subsoil horizons. We determined soil depth by sampling a soil core in the center of the study plots. We used a motor driven soil column cylinder with a diameter of 8.3 cm for the soil sampling (Eijkelkamp, Giesbeek, The Netherlands). For soil pH and clay content, a composite sample representing the soil of the whole plot was prepared by mixing 14 mineral topsoil samples (0-10 cm, using a manual soil corer with 5.3 cm diameter) from the same plot. Soil samples were air dried and sieved (< 2 mm), and we then measured the soil pH in the supernatant of a 1:2.5 mixture of soil and 0.01 M CaCl2.

All collected samples were operated in the field and sorted in the lab by trained technicians. Species identification was done by expert taxonomists. All people involved in data collection are listed in the acknowledgments section.

Timing and spatial scale

The timing of the sampling was selected to coincide with the annual peak of biological activity for each of the different trophic groups:
- Primary producers were sampled annually from mid-May to mid-June, from 2008 to 2018.
- Aboveground fungal pathogens were sampled from July to August, in 2011.
- Molluscs were sampled in June 2017.
- Aboveground arthropods were sampled from May to August in 2008.
- Birds and bats were sampled annually from March to June, from 2008 to 2012.
- Belowground AM fungal symbionts, fungal pathogens, fungal decomposers, bacterial decomposers, protistan bacterivores, protistan parasites, protistan omnivores were sampled from July to August 2011, 2014 and 2017.
- Belowground insect herbivores and arthropod predators were sampled in Spring 2011.
- Belowground arthropod decomposers were sampled in Spring 2008 to 2011, and in Spring 2019.

Within the Biodiversity Exploratories project, some taxonomic and functional groups are sampled regularly by 'core' projects (e.g. vascular plants), while the sampling and data gathering on other groups depend on the funding of more temporary 'contributing' projects. Therefore, all groups have not been sampled annually.

All these data were collected within a 50 m x 50 m area, in 150 grassland plots. These grassland plots were chosen to cover a wide gradient of land-use intensity.

To assess the surrounding plant diversity of each grassland plot, we have also surveyed the vegetation within the major surrounding homogeneous vegetation zones in a 75-m radius of each plot from May to July (during the growing season) in 2017 and 2018. These zones were mostly situated within the same grassland-field as the focal plot but we occasionally surveyed other habitat types (c. 20% were situated in hedgerows, margins or forests).

	The landscape composition was determined in 2008 in a 2000-m radius of the center of the plot.
Data exclusions	No data was excluded from the analyses
Reproducibility	There are no experiments in the study. Our data were collected as part of a monitoring over several years and which cannot be repeated.
Randomization	Study plots were selected from 3000 candidate plots. Surveys of initial vegetation and land use were conducted on candidate plots by stratified random sampling to ensured that the selected plots covered the whole range of land-use intensity and to minimize confounding effects of spatial position or soil type.
Blinding	Investigators were not aware of the land-use intensity of the plot were they worked, but they could not otherwise be blinded during data collection and analyses for example with respect to the year a sample came from.

Did the study involve field work? ☒ Yes ☐ No

Field work, collection and transport

Field conditions	For aboveground arthropods, the sampling was carried out during the day, when the vegetation was dry (no rainfall) and wind speed was low.<br><br>For birds, the sampling was carried out during the morning chorus (sunrise-11:00h) when the wind speed was low. In exceptional cases, observations were made during the evening chorus (last 3 hours before sunset).<br><br>For all other organisms, the sampling was operated at all weather conditions.
Location	Our data were collected in three German regions: (1) Schwäbische Alb in south-western Germany (420 km², 460–860 m above sea level (a.s.l.), Latitude: 48.413, Longitude: 9.4912); (2) Hainich-Dün in central Germany (1560 km², 285–550 m a.s.l., Latitude: 51.1186, Longitude: 10.5056); and (3) Schorfheide-Chorin in northeastern Germany (1300 km², 3–140 m a.s.l., Latitude: 53.0178, Longitude: 14.0042). Exact plot locations cannot be disclosed due to a legal agreement with landowners.
Access & import/export	Fieldwork permits were issued from 2008 to 2021 by the responsible state environmental offices of Baden-Württemberg (Regierungspräsidium Tübingen), Thüringen (Thüringer Landesverwaltungsamt) and Brandenburg (Landesumweltamt Brandenburg).
Disturbance	Activity of investigators was spatially limited to the 50 m x 50 m grassland plots. We used small paths to access the plots and carefully ensure to avoid any damage to the habitat. Destructive sampling was minimized by using protocols described in the Methods section.

Reporting for specific materials, systems and methods

We require information from authors about some types of materials, experimental systems and methods used in many studies. Here, indicate whether each material, system or method listed is relevant to your study. If you are not sure if a list item applies to your research, read the appropriate section before selecting a response.

Materials & experimental systems

n/a	Involved in the study	
☒	☐	Antibodies
☒	☐	Eukaryotic cell lines
☒	☐	Palaeontology and archaeology
☐	☒	Animals and other organisms
☒	☐	Human research participants
☒	☐	Clinical data
☒	☐	Dual use research of concern

Methods

n/a	Involved in the study	
☒	☐	ChIP-seq
☒	☐	Flow cytometry
☒	☐	MRI-based neuroimaging

Animals and other organisms

Policy information about studies involving animals; ARRIVE guidelines recommended for reporting animal research

Laboratory animals	No laboratory animals were involved in the study.
Wild animals	In order to quantify the diversity of belowground microorganisms, soil samples were collected in the field and then transport to the lab were the DNA or RNA was isolated from soil and sequenced. Arthropods were collected in the field and killed using ethanol. Identification of arthropods requires killing and transport to the lab were microscopes can be used. Molluscan samples were collected in plastic bags and transferred to the laboratory. Afterward, snail shells were collected by hand using a stereomicroscope. Vertebrate species were assessed by remote observation only (see Methods section for details).
Field-collected samples	Aboveground arthropod samples were stored in 93% ethanol at 7°C except for short time periods during transport, sorting and identification. Molluscan samples were collected in plastic bags, transferred to the laboratory, and dried for 48 h at 40°C. Afterward, snail shells were collected by hand using a stereomicroscope.

	Belowground insect herbivores', arthropod predators' and arthropod decomposers' samples were stored in plastic cups and transported as soon as possible to the laboratory in cooler boxes (10°C), extracted in 70% diethylene glycole and stored in 70 % ethanol. Soil samples for DNA extraction were transported in cooled boxes (approx. 4°C - 8°C) to the field lab where within few hours after soil excavation the soil was sieved and roots were removed. These samples were then frozen at -20°C. Soil samples for RNA extraction were flash-frozen directly after excavation either in liquid nitrogen or dry ice. Soil samples were shipped from the field labs to the institute laboratories on dry ice. Until nucleic acid extraction, soil samples were stored either at -20°C (for DNA extraction) or liquid nitrogen (for RNA extraction). The DNA and RNA nucleic acid extracts were stored at -20°C or -80°C, respectively.
Ethics oversight	It could not be ruled out that threatened or protected species would be collected and killed. Thus, permision was required from the authorities which was granted for scientific reasons. These permits were issued by the responsible state environmental offices of Baden-Württemberg (Regierungspräsidium Tübingen), Thüringen (Thüringer Landesverwaltungsamt) and Brandenburg (Landesumweltamt Brandenburg).

Note that full information on the approval of the study protocol must also be provided in the manuscript.
